# Transcriptomic analyses reveal species-specific light-induced anthocyanin biosynthesis in chrysanthemum

**DOI:** 10.1186/s12864-015-1428-1

**Published:** 2015-03-17

**Authors:** Yan Hong, Xingjiao Tang, He Huang, Yuan Zhang, Silan Dai

**Affiliations:** College of Landscape Architecture, Beijing Forestry University, No. 35 Tsinghua East Road, Beijing, 100083 China

**Keywords:** Anthocyanin, Bioinformatics, Capitulum development, *Chrysanthemum* × *morifolium*, Flower colour, Light induction, Transcriptome

## Abstract

**Background:**

The flower colour of agricultural products is very important for their commercial value, which is mainly attributed to the accumulation of anthocyanins. Light is one of the key environmental factors that affect the anthocyanin biosynthesis. However, the deep molecular mechanism remains elusive, and many problems regarding the phenotypic change and the corresponding gene regulation are still unclear. In the present study, *Chrysanthemum* × *morifolium* ‘Purple Reagan’, a light-responding pigmentation cultivar, was selected to investigate the mechanism of light-induced anthocyanin biosynthesis using transcriptomic analyses.

**Results:**

Only cyanidin derivatives were identified based on the analyses of the pigmentation in ray florets. Shading experiments revealed that the capitulum was the key organ and that its bud stage was the key phase responding to light. These results were used to design five libraries for transcriptomic analyses, including three capitulum developmental stages and two light conditions. RNA sequences were *de novo* assembled into 103,517 unigenes, of which 60,712 were annotated against four public protein databases. As many as 2,135 unigenes were differentially expressed between the light and dark libraries with 923 up-regulated and 1,212 down-regulated unigenes in response to shading. Next, interactive pathway analysis showed that the anthocyanin biosynthetic pathway was the only complete metabolic pathway both modulated in response to light and related to capitulum development. Following the shading treatment, nearly all structural genes involved in the anthocyanin biosynthetic pathway were down-regulated. Moreover, three *CmMYB* genes and one *CmbHLH* gene were identified as key transcription factors that might participate in the regulation of anthocyanin biosynthesis under light conditions based on clustering analysis and validation by RT-qPCR. Finally, a light-induced anthocyanin biosynthesis pathway in chrysanthemums was inferred.

**Conclusion:**

The pigmentation of the ray florets of chrysanthemum cultivar ‘Purple Reagan’ is dependent on light. During the light-induced pigmentation process, the expression of seven structural genes in the anthocyanin biosynthetic pathway (regulated by at least four transcription factors in response to light) are the main contributors to the pigmentation of chrysanthemums. This information will further our understanding of the molecular mechanisms governing light-induced anthocyanin biosynthesis in ornamental plants.

**Electronic supplementary material:**

The online version of this article (doi:10.1186/s12864-015-1428-1) contains supplementary material, which is available to authorized users.

## Background

The colours of flowers and fruits of agricultural products are highly important for their commercial value, which are mainly attributed to the accumulation of anthocyanins, a class of plant flavonoid metabolites [1]. The genetics and biochemistry of the anthocyanin biosynthetic pathway have been well characterized in plants, such as arabidopsis (*Arabidopsis thaliana*), snapdragon (*Antirrhinum majus*) and petunia (*Petunia hybrida*) [2]. Anthocyanin biosynthetic pathway genes are also regulated by the developmental stages of plants and environmental factors [3]. However, the mechanisms associated with plant development and/or environmental factors that impact anthocyanin biosynthesis remain unclear.

Light is one of the key environmental factors that affect anthocyanin biosynthesis [4]. Previous studies demonstrated that light has a decisive effect on colour production in plants; thus, the ornamental values of flowers and economic values of fruits can be greatly improved by changing their colours through the regulation of light conditions [5]. The pigmentation of reproductive organs can be divided into the following two categories according to the different modes of light regulation on plant species: the light-dependent type [6-9] and the light-independent type [10,11]. Many studies have demonstrated that the expression of anthocyanin genes was induced by intense light, resulting in the accumulation of anthocyanin. In contrast, under weak light or dark conditions, the expressions of related genes were down-regulated or repressed, which resulted in decreased accumulation of anthocyanin in flowers and fruits, thereby generating white or light-coloured organs [9,11-13]. Moreover, many regulatory genes have been identified in different crop species, for example, *MdCOP1* and *MdMYB1* in apple (*Malus pumila*) [8,14], and *RLC1* in cotton (*Gossypium hirsutum*) [7]. However, the deep molecular mechanism of light-induced anthocyanin biosynthesis remains elusive, and many problems regarding the phenotypic change and corresponding gene regulation are still unclear in ornamental plants.

Chrysanthemum (*Chrysanthemum* × *morifolium*) is a worldwide famous ornamental crop with rich germplasm, whose yield and output values make it a leader in the global flower industry [15,16]. Compared with other ornamental traits, the phenotypic variation of chrysanthemum flower is particularly rich in colours [17]. Previous studies have shown that only one pathway related to anthocyanin metabolism (the cyanidin metabolic pathway) exists in the chrysanthemum [18,19]. The simple background of pigment metabolism and the light sensitivity of pigments production make chrysanthemums an ideal model for studies of anthocyanin biosynthesis and the corresponding molecular regulatory mechanism in response to light.

Next generation sequencing technology, such as high-throughput paired-end (PE) RNA sequencing (RNA-Seq) and digital transcript abundance tag profiling, has greatly facilitated investigation of the functional complexity of transcriptomes for non-model organisms without a reference genome [20-23]. *De novo* assembly of a transcriptome from RNA-Seq data produces a genome-scale transcription map that contains both the transcriptional structure and expression level for each gene. This approach has been widely used for studies on the relationship between environmental factors and plant colour changes [21,23-28].

To further understand the mechanism behind light-induced pigmentation in the chrysanthemum, in the present study, the ray florets of chrysanthemum cultivar ‘Purple Reagan’ were analysed using transcriptomic methods. To the best of our knowledge, this is the first systematic study to investigate light-induced anthocyanin biosynthesis using these two strategies in ornamental plants. This information will be very helpful for improving functional genomic studies in chrysanthemums and will further our understanding of the molecular mechanisms behind light-induced anthocyanin biosynthesis. Furthermore, this study provided evidence for a molecular breeding theory concerning the basis of flower colour modifications in ornamental plants.

## Methods

### Plant materials and RNA preparation

The capitulum of chrysanthemum cultivar ‘Purple Reagan’ was selected as the experimental material. This cultivar is pentaploid (2n=5x=45), with the floral competence of 14-leave stage, and the limited inductive photoperiod of 43 d under short daylight (12 h light/12 h dark) (unpublished data). According to Sun et al. [19], a total of five stages have been defined during capitulum development, named S1, S2, S3, S4 and S5. Ray floret samples were collected at each capitulum developmental stage under light (fluorescent lamp) and dark (shading using silver paper over the whole capitulum) conditions in September 2013 from an artificial chamber located at the Beijing Forestry University, Beijing, China. All samples were frozen rapidly in liquid nitrogen and kept at −80°C. A portion of the samples was used for RNA extractions.

### Phenotypic measurements

Ray floret colour during different capitulum developmental stages was measured using a Hunter Lab Mini Scan XE Plus colourimeter (Hunter Associates Laboratory Inc., Tucson, AZ, USA). The Commission Internationale de l’Eclairage L*a*b* colour scale was adopted [17], and raw data such as L*, a* and b* were obtained. The colour index for red grapes (CIRG) was calculated according to CIRG = (180-*H*) / (L* + *C*), where *C* = (a*^2^ + b*^2^)^0.5^ and *H* = arctan (b*/a*) [29]. A vernier caliper was used to measure capitulum diameters. Five random measurements were made for each sample; the mean value was used for analysis.

### Measurements for high performance liquid chromatography and reducing sugar content

The extraction of anthocyanins was performed according to previous methods [19]. Briefly, 0.2 g of the sample was ground into fine powder in liquid N_2_, and then homogenized in 1 ml of anthocyanin extracts [methanol:distilled water:methane acid:trifluoroacetic acid (70:27:2:1, v/v/v/v)] at 4°C for 24 h, with vortexing every 6 h [10,30]. Then, the mixture was filtered using medium-speed filter paper (Hangzhou Special Paper Industry, Hangzhou City, China), and the filtrate was passed through a 0.22 μm reinforced nylon membrane filter (Shanghai ANPEL, Shanghai City, China) before submitting to high performance liquid chromatography-diode array detector (HPLC-DAD) analysis. Three replicate extractions were made for each biological sample. The HPLC system Dionex (Thermo Fisher Scientific Inc, Sunnyvale, CA, USA) equipped with a P680 HPLC pump, UltiMate 3000 autosampler, Thermostatted Column Compartment-100 and Photodiode Array Detector-100 was used to separate the constituents of the ray floret extracts. A C18 ODS-80Ts QA column (150 × 4.6 mm I.D., Tokyo, Japan) protected with a CARB Sep Coregel 87C guard cartridge (Transgenomic Inc., Omaha, NE, USA) was used. A 10 mm^3^ aliquot was injected, and the resulting chromatograms were read at 515 nm for anthocyanins. For preparation of the standard solution, rutin was accurately weighed and dissolved in methanol, and then diluted to appropriate concentrations. The quantitative analysis was based on the method described by Lin and Harnly [31]. Each sample run for HPLC was repeated three times under the same conditions. The measurement for reducing sugar content was based on Lin [32].

### RNA isolation and library construction for transcriptomic analyses

Total RNA was isolated from the ray florets of the capitulum during different developmental stages and light conditions using the Quick RNA Isolation Kit (Huayueyang Biotechnology Co. Ltd., Beijing, China). To obtain a general overview of the ray floret transcriptome in response to light, five libraries (L1, L2, L3, D2 and D3) were designed for RNA-Seq. L1, L2 and L3 represent samples that were treated under a fluorescent lamp and were sampled at capitulum developmental stages S1, S2 and S3, respectively; D2 and D3 represent samples that were 100% shaded using silver papers at the S1 stage and were sampled once every other 48 h when they developed to S2 and S3 stages, respectively (Additional file 1: Figure S1).

After the total RNA extraction and DNA polymerase I treatment, magnetic beads with Oligo (dT) were used to isolate mRNA. The mRNA was mixed with the fragmentation buffer and fragmented into short fragments. Then, cDNA was synthesized using the mRNA fragments as templates. Short fragments were purified and resolved with elution buffer for end reparation and single nucleotide adenine addition. Next, the short fragments were connected with adapters. Suitable fragments were selected for PCR amplification as templates. During the quality control steps, an Agilent 2100 Bioanaylzer (Agilent Technologies Co. Ltd., Santa Clara, CA, USA) and an ABI StepOnePlus Real-Time PCR System (Applied Biosystems Inc., Foster, CA, USA) were used.

### Transcriptome sequencing, *de novo* assembly and functional annotation

The Illumina HiSeq™ 2000 (Illumina Inc., San Diego, CA, USA) was employed to sequence the library. The cDNA fragments were approximately 200 bp in length, and the fragments were sequenced using the PE strategy. The raw reads obtained were pre-processed by removing adaptor sequences and discarding empty reads and low-quality sequences. Then, all reads were used for transcriptome *de novo* assembly using the short read assembling program SOAPdenovo version 1.04 (http://soap.genomics.org.cn/soapdenovo.html) with the parameters “-K 29, −M 2, −L 50”. The meaning and selection principles of the parameters are available at http://soap.genomics.org.cn/soapdenovo.html. Short reads were first assembled into contigs with no gaps, and the reads were mapped back to the contigs. The resulting contigs were joined into scaffolds using the read mate pairs, with unknown sequences replaced with “N”s. Finally, PE reads were performed to fill the gaps between different scaffolds to obtain unigenes with the least number of “N”s and the longest sequences. The assembled unigenes were annotated using the BLASTx alignment (E-value < 1×10^−5^) to protein databases, such as the National Center for Biotechnology Information non-redundant (NCBI nr) protein database (http://www.ncbi.nlm.nih.gov), the Swiss-Prot protein database (http://www.expasy.ch/sprot), the Kyoto Encyclopedia of Genes and Genomes (KEGG) pathway database (http://www.genome.jp/kegg), and the Clusters of Orthologous Groups of proteins (COG) database (http://www.ncbi.nlm.nih.gov/COG). The best-aligning results from the four databases were chosen to decide the sequence direction of the unigenes.

### Expression annotation

The alignment package SOAPaligner version 2.20 was used to map reads back to the transcriptome with the parameters “-m 0, −× 1000, −s 40, −l 35, −v 3, −r 2”. The meaning and selection principles of the parameters are available at http://soap.genomics.org.cn/soapaligner.html. Then, the number of mapped clean reads for each unigene was counted and normalized into fragments per kilobase per million fragments (FPKM) values, which are widely used to calculate unigene expression [33]. In our work, the differentially expressed unigenes between each of the samples were screened with a threshold of false discovery rate (FDR) < 0.001 and an absolute value of log_2_ ratio ≥ 1 [34].

### Metabolic pathway analyses

Interactive pathway (iPath) analysis was performed using Interactive Pathways Explorer version 2.0 (http://pathways.embl.de). The expression of a specific gene family was summed from all family members encoding the gene based on KEGG orthology (Ko) identities. To understand the dynamic changes and magnitude of absolute expression during capitulum development, a bar consisting of different colours was applied to indicate different FPKM values of unigenes.

### Homolog search and phylogenetic tree construction

A total of 34 *R2R3-MYB* and 25 *bHLH* genes were isolated from 103,517 chrysanthemum unigenes and translated using the NCBI Open Reading Frame Finder (http://www.ncbi.nlm.nih.gov/projects/gorf). Sequences of all 125 arabidopsis R2R3-MYB proteins were retrieved from The Arabidopsis Information Resource Arabidopsis Genome Annotation version 7.0 released in April 2007 (http://www.arabidopsis.org). Additionally, 149 *AtbHLH* sequences were retrieved from the UniProt Database (http://www.uniprot.org). Sequence alignments were performed using the ClustalW algorithm-based AlignX mode in Molecular Evolutionary Genetics Analysis version 5 (MEGA5) [35]. A phylogenetic tree was subsequently constructed according to the neighbour-joining statistical method [36]. Tree nodes were evaluated using the bootstrap method for 1,000 replicates [37], and branches corresponding to partitions reproduced in less than 50% of the bootstrap replicates were condensed into single branches. Evolutionary distances were computed using the *p*-distance method and expressed in units of amino acid differences per site. All positions containing gaps and missing data were eliminated prior to construction of the phylogenetic trees for the *R2R3-MYB* and *bHLH* genes. Amino acid sequences of the R2R3-MYB and bHLH proteins in chrysanthemums and other species were aligned in MEGA5 [35]. The conserved motifs of each R2R3-MYB subgroup (Sg) were predicted using MEME (http://nbcr-222.ucsd.edu).

### Real-time quantitative reverse transcription-PCR analysis

For expression analysis using real-time quantitative reverse transcription-PCR (RT-qPCR), RNA was isolated from the five different developmental stage samples under light and dark conditions and used to generate cDNA. PCR reactions were performed using a Mini Opticon Real-time PCR System (Bio-Rad Laboratories Inc., Hercules, CA, USA) based on the SYBR Premix Ex Taq (TaKaRa Bio Inc., Shiga, Japan) [38] with three replicates. The mean expression levels of different genes were normalized to the relative expression level [38]. Primer sequences are listed in Additional file 2: Table S1.

## Results and discussion

### Pigments content in the ray florets of chrysanthemum cultivar ‘Purple Reagan’

**Figure 1:**
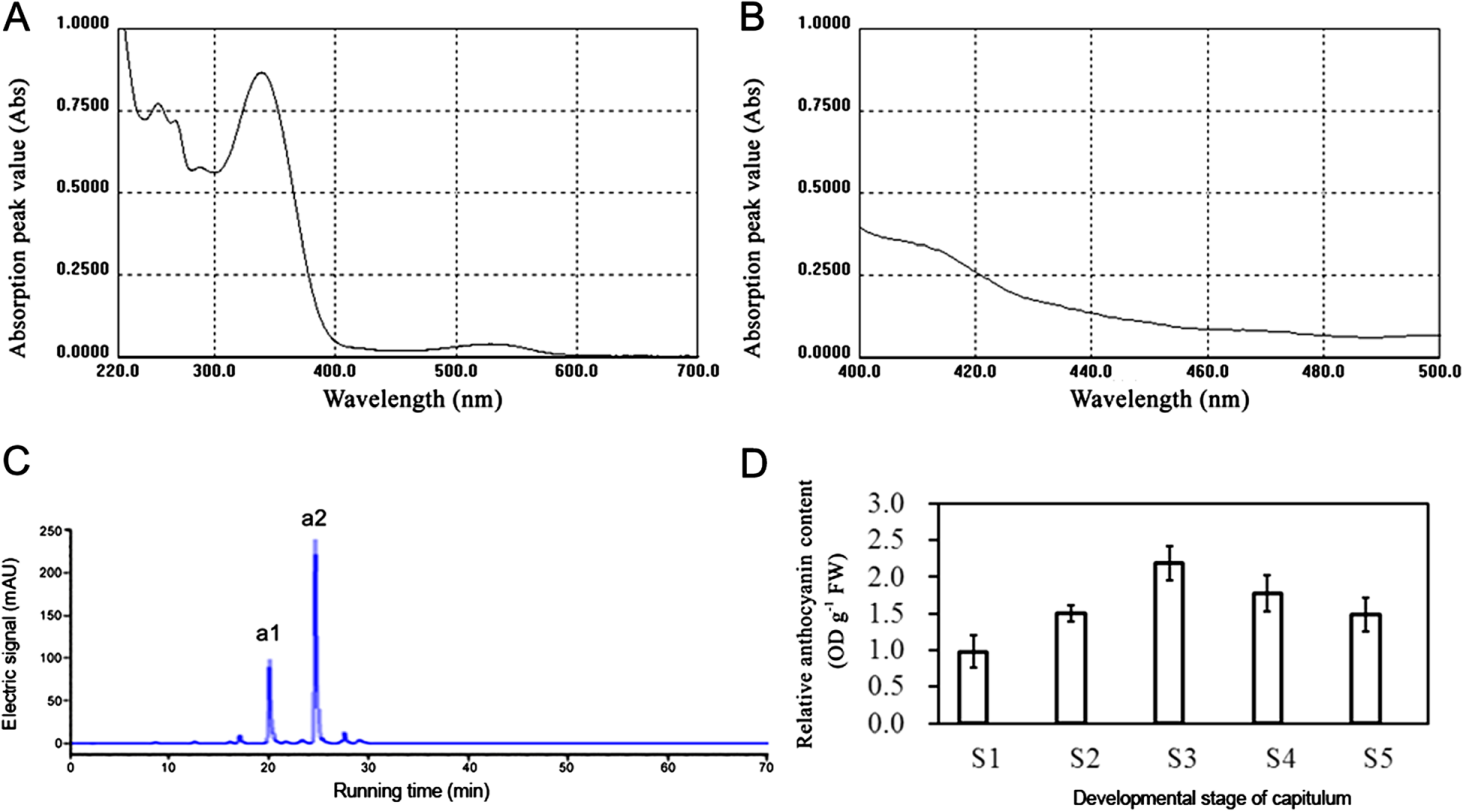
**Analysis of the ultraviolet–visible spectrum and relative anthocyanin content in** ***Chrysanthemum*** **×** ***morifolium*** **‘Purple Reagan’. A**, Ultraviolet–visible spectrum analysis of flavonoids. **B**, Ultraviolet–visible spectrum analysis of carotenoids. **C**, Mass spectrum analysis of anthocyanin content using the HPLC method. **D**, The relative anthocyanin contents of different samples collected during five capitulum developmental stages detected by the HPLC method. The x-axis and y-axis in Figure 1A and B indicate the wavelength and absorption peak value, respectively. The x-axis and y-axis in Figure 1C indicate the running time and electric signal, respectively. The x-axis and y-axis in Figure 1D indicate the five capitulum developmental stages from S1 to S5 and the relative anthocyanin content, respectively.

The pigment types and contents in flowers result in the variety of flower colours. Therefore, we investigated the pigment types that existed in the ray florets of chrysanthemum cultivar ‘Purple Reagan’. Spectral scanning of ray floret extracts from 220 nm to 270 nm and 400 nm to 700 nm using the ultraviolet–visible spectrum revealed that the absorption only presented between 220 nm and 400 nm and between 500 nm and 600 nm. This result indicated that the analysed samples contained only anthocyanin and flavonoids (Figure 1A and B). The anthocyanin content was further measured using HPLC-multi stage tandem mass spectrometry. The measuring result showed that the fractionations of the a1 and a2 peaks both included a fragment of m/z 287 u, typical for a [cyanidin]^+^ ion (Figure 1C). Based on Nakayama et al. [18] and Saito et al. [39], peaks a1 and a2 were assigned as cyanidin-3-*O*-(6″-*O*-malonyl-glucoside) and cyanidin-3-*O*-(3″,6″-*O*-dimalonyl-glucoside), respectively. Quantitative analysis was further conducted using samples from the five capitulum developmental stages S1 to S5 using HPLC compared to the standard sample. As shown in Figure one (D), relative anthocyanin contents were increased during capitulum developmental stages S1 to S3 and then decreased after the S3 stage. Therefore, the change in flower colour phenotypes in chrysanthemums is mainly caused by the increase or decrease of anthocyanin (cyanidin) content.

### Organs responding to light during the anthocyanin biosynthetic process

**Figure 2:**
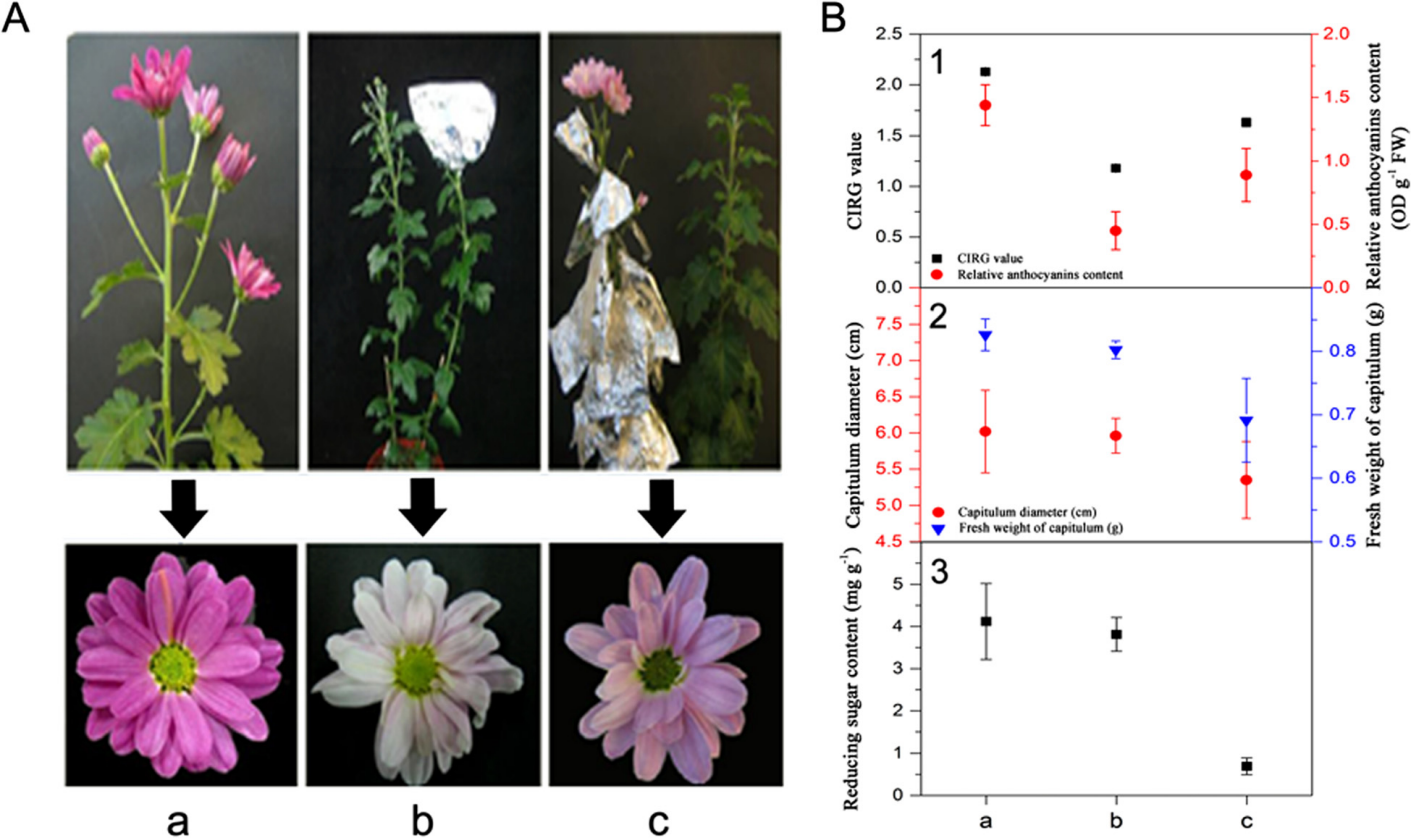
**Schematic of phenotypic and physiological data among different shading treatments. A**, The different shading treatments and corresponding ray floret phenotypes (S5 developmental stage of capitulum). **B**, Comparison of CIRG values and relative anthocyanin contents in ray florets (B-1), capitulum diameters and fresh weights of capitulum (B-2), and reducing sugar contents (B-3) in ray florets (S5 developmental stage of capitulum) following different shading treatments. a, b and c represent the control group (non-shaded treatment), capitulum-shaded and leaf-shaded treatment, respectively.

To investigate whether the ray florets or the leaves are the light-response receptors during the anthocyanin biosynthetic process in chrysanthemums, we first shaded the whole capitulum during the S1 stage (bud stage) and all leaves using silver papers; then, we took photos when the capitulum developed to the S5 stage (full-bloom stage, Figure 2A). Plants under light conditions were used as a control group. As shown in Figure two (A), compared with the control group, the ray florets under capitulum- and leaf-shaded treatments all faded; the former treatment made the fading more severe.

Based on the fading phenomenon, the CIRG and relative contents of total anthocyanin in the ray florets between these two treatments were further measured and compared. Compared with the control group, the CIRG value and relative anthocyanin contents in the ray florets under these two treatments were all decreased (Figure 2B-1). The decreased amount of relative anthocyanin content under the capitulum-shaded treatment (0.99 OD g^−1^ FW) was greater than the leaf-shaded treatment (0.55 OD g^−1^ FW), indicating that the shading treatment during the bud stage of the chrysanthemum capitulum significantly affected the relative content of total anthocyanin.

**Figure 3:**
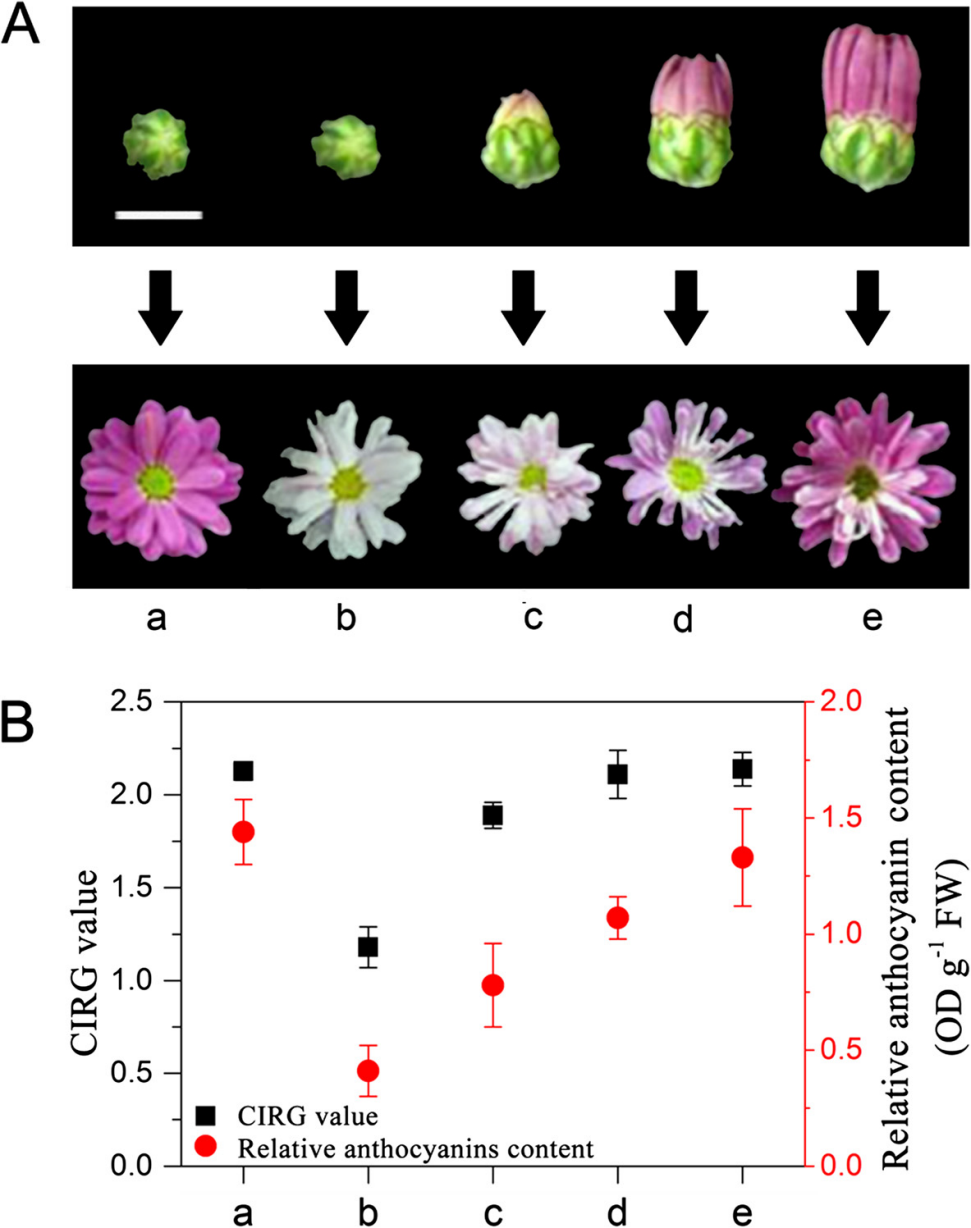
**Schematic of phenotypic and physiological data among different shading treatments of capitulum developmental stages. A**, The different developmental stages of the capitulum before treatment and the corresponding ray floret phenotypes at the S5 developmental stage of the capitulum. Scale bar = 1 cm. **B**, Comparison of CIRG values and relative anthocyanin contents in ray florets (S5 developmental stage of the capitulum) among different treatments. a represents the control group (capitulum treated under light condition from stage S1 until stage S5), while b, c, d and e represent capitulum treated under dark conditions from stages S1 to S4, respectively, until developed to stage S5.

The effect of shading on capitulum development was also investigated between these two treatments by measuring the capitulum diameters (S5 stage), fresh weights and reducing sugar contents in ray florets. The results showed that the capitulum diameters were decreased following the two shading treatments to different extents compared to the control group (Figure 2B-2). Variance analysis indicated that the decrease in capitulum diameters between the control group and the capitulum-shaded treatment was not significant; in contrast, the opposite was true for the leaf-shaded treatment (Figure 2B-2). The measuring results also showed that the fresh weights and reducing sugar contents in ray florets decreased significantly following shading of the leaves (Figure 2B-2 and B-3). The above results indicated that capitulum development of chrysanthemums was significantly repressed by shading the leaves.

Based on measurements of flower colour phenotypes, pigments and related physiological substances, we inferred that the capitulum might be the key organ responding to light during the anthocyanin biosynthetic process.

### Key phase of capitulum development responding to light during the anthocyanin biosynthetic process

To identify the developmental stage of capitulum that acts as the key phase in response to light during the anthocyanin biosynthetic process, we shaded the capitulum during four of the developmental stages (S1, S2, S3 and S4) and found that the colour phenotypes of ray florets under each capitulum developmental stage were all faded compared to the control group. The phenotypic change in ray floret colour following shading during the S1 stage was most obvious, resulting in ray florets that faded to almost white (Figure 3A). The CIRG values and relative contents of total anthocyanin in the ray florets under the four shading treatments all decreased (Figure 3B). The decreased amount of relative anthocyanin content in ray florets during the S1 stage was the highest (1.03 OD g^−1^ FW), and there was a concomitant decrease in anthocyanin content in ray florets from stages S2 to S4 (Figure 3B).

In conclusion, the pigmentation of chrysanthemum ray florets was significantly repressed after shading during the early capitulum developmental stage, indicating that the early developmental stage of the capitulum was the key phase that responds to light. This result was consistent with an investigation of the Barberton daisy (*Gerbera jamesonii*) [40]. Therefore, we inferred that during the early capitulum developmental stages, light plays a signal-activating role in the anthocyanin biosynthetic process or induces the generation of physiological substances in the capitulum that are necessary for flower development and pigmentation. In contrast, the later capitulum developmental stages were only indistinctively dependent on light.

### Transcriptome sequencing and *de novo* assembly

A pooled cDNA sample representing the capitulum developmental stages of the chrysanthemum was prepared and sequenced with Illumina PE sequencing. As shown in Additional file 3: Table S2, after a stringent quality check and data cleaning, all PE reads were assembled into 103,517 unigenes. The quality of the reads was assessed using the base-calling quality scores from Illumina’s Base-caller Bustard. Additionally, 97.53% (D3) to 97.71% (L2) of the clean reads had Phred-like quality scores at the Q20 threshold (percentage of sequences with sequencing error rates lower than 1%), which was higher than the 88% and 94.85% reported for similar works investigating the flowers from tea (*Camellia sinensis*) [41] and chamomile (*Chrysanthemum lavandulifolium*) [42], respectively. The proportion of guanidine and cytosine nucleotides among the total nucleotides ranged from 43.08% (D2) to 43.61% (D3). These results showed that the throughput and sequencing quality was high enough for further analysis.

Using the SOAPdenovo software (http://soap.genomics.org.cn), a total of 151,247, 154,112, 140,788, 156,237 and 145,369 contigs (≥200 bp) were assembled into samples L1, L2, L3, D2 and D3, respectively (Additional file 3: Table S2; Sheet A in Additional file 4: Table S3). Using PE joining and gap-filling, a total of 93,998, 96,119, 83,946, 93,496 and 83,467 unigenes (≥200 bp) were further assembled from these contigs respectively (Additional file 3: Table S2; Sheet B in Additional file 4: Table S3 and Additional file 5: Figure S2). Finally, a total of 103,517 unique unigenes were screened out, which was similar to the study of the Barberton daisy [25].

### Functional annotation and classification

A total of 60,712 unigenes (58.65% of all 103,517 cleaned unigenes) were annotated based on BLASTx (E-value < 1×10^−5^) searches of four public databases: NCBI nr database, Swiss-Prot protein database, KEGG database and COG database; this was higher than the percentage reported for the chamomile transcriptome (54.32%) [42]. Among them, 57,952 unigenes (95.45% of all annotated unigenes) could be annotated to the NCBI nr database, while 16,359 unigenes could be annotated to all four databases (Additional file 6: Figure S3A). Based on the NCBI nr annotations, 40.9% of the annotated sequences had very strong homology (E-value < 10^−60^), 18.6% showed strong homology (10^−60^ < E-value < 10^−30^), and an additional 40.3% showed homology (10^−30^ < E-value < 10^−5^) to available plant sequences (Additional file 6: Figure S3B). The 20 top-hit species based on NCBI nr annotation are shown in Additional file 6: Figure S3C. More than 80% of unigenes could be annotated with sequences from the nine top-hit species.

The Swiss-Prot database was reviewed by manual endorsement; therefore, the functional annotation that was provided by this database was the least redundant, the most complete and highly accurate. In total, 38,215 unigenes were annotated against the Swiss-Prot database, accounting for 62.94% of all annotated unigenes and 246 unigenes that were not annotated against the other three databases (Additional file 6: Figure S3A).

There were 34,657 unigenes mapped into 128 KEGG pathways. The pathways with the highest unigene representations were plant-pathogen interaction (Ko04626, 1,804 unigenes, 5.21%), followed by plant hormone signal transduction (Ko04075, 1,567 unigenes, 4.52%) and RNA transport (Ko03013, 1,352 unigenes, 3.9%). The metabolites with the highest representation were starch and sucrose metabolism (Ko00500, 916 unigenes, 2.64%), purine metabolism (Ko00230, 825 unigenes, 2.38%), and the mRNA surveillance pathway (Ko03015, 802 unigenes, 2.31%) (Additional file 7: Table S4). From these pathways, information concerning chrysanthemum metabolism can be obtained. Additionally, the Ko ids were used in iPath analysis and were helpful for the study of flower colour quality-related metabolism (see below).

Next, Gene Ontology (GO) analysis was performed. A total of 42,411 unigenes (69.86% of all annotated unigenes) were assigned one or more GO terms. These 42,411 unigenes were categorized into 55 GO functional groups distributed under three main categories: biological processes (49.53%), cellular components (36.67%) and molecular functions (13.80%). Proteins related to cellular processes (15.48%), metabolic processes (14.60%) and single-organism processes (11.12%) were enriched in the biological processes category. Under the cellular components category, the cell (24.76%), cell parts (24.76%) and organelles (19.81%) were the most highly represented GO terms. Within the molecular functions category, genes encoding binding proteins (41.68%) and proteins related to catalytic activity (43.92%) were the most enriched (Additional file 8: Figure S4).

In addition to GO analysis, COG analysis was used to further evaluate the function of the assembled unigenes. Out of a total of 60,712 annotated unigenes, 20,414 hits (33.62%) were aligned with 25 COG classifications. Out of the 25 COG categories, the cluster for general function prediction only (16.51%) represented the largest group, followed by transcription (9.34%) and replication, recombination and repair (8.64%). Only a small proportion of the unigenes was assigned to nuclear structure (0.027%) or extracellular structures (0.027%). It is worth noting that a large number of genes were assigned to posttranslational modification, protein turnover, chaperones (7.44%), translation, ribosomal structure and biogenesis (6.95%), signal transduction mechanisms (6.54%), and carbohydrate transport and metabolism (5.79%) (Additional file 9: Figure S5).

Based on the use of BLASTx against the databases mentioned above, the direction and region of the coding sequences were extracted from the sequences. A total of 58,419 coding sequences were translated into peptide sequences; a total of 22,641 had lengths > 300 AA, with the 888 longest unigenes having lengths > 1000 AA. The remaining unigenes were predicted using ESTscan [43] resulting in annotation of 4,715 unigenes, 589 of whose lengths were > 300 AA.

### Exploration of light-induced genes during capitulum development

A total of 103,517 unigenes expressed during capitulum development showed different expression patterns between the light and dark libraries. For a global view of expression patterns, the expression level of unigenes was visualized in a three-dimensional space (Additional file 10: Figure S6). This approach enables an overall understanding of the expression changes of unigenes between these two libraries.

To investigate differences in unigene expression in chrysanthemum ray florets under different light conditions, we first analysed the differentially expressed unigenes between the light and dark libraries. Comparing the expression of unigenes between samples L2 (under light conditions) and D2 (under dark conditions), we found that a total of 2,425 and 2,542 unigenes were up- and down-regulated after shading, respectively (Additional file 11: Figure S7A and S7C). For unigenes in samples L3 (under light conditions) and D3 (under dark conditions), a total of 2,807 and 2,514 unigenes were up- and down-regulated, respectively (Additional file 11: Figure S7B and S7C). To identify candidate unigenes that are regulated in response to light, the intersections among these four samples were further analysed. Finally, we found that the numbers of up-regulated, down-regulated and invariable unigenes were 923, 1,212 and 93,656, respectively (Additional file 11: Figure S7C). Thus, we focused on the 923 up-regulated and 1,212 down-regulated unigenes that most likely represented light-induced genes.

To identify the function of these differentially expressed unigenes, we further performed GO, KEGG and nr classification analysis. In the GO analysis, these differentially expressed unigenes were categorized into 47 functional groups. Interestingly, unigenes encoding proteins related to biological adhesion, antioxidant activity and extracellular region were only up-regulated, while unigenes encoding proteins related to the extracellular matrix and nutrient reservoir activity were only down-regulated (Additional file 12: Figure S8A). In the KEGG analysis, all of the differentially expressed unigenes were categorized into 12 functional groups. Among them, 60.19% of the up-regulated unigenes and 68.18% of the down-regulated unigenes with known functions fell into categories related to genetic information processing, carbohydrate metabolism, flavonoid related biosynthesis, hormone response, amino acid metabolism or environmental adaptation (Additional file 12: Figure S8B), suggesting that these pathways or processes might respond to shading.

Because GO categories and KEGG annotation are too general to provide detailed information on biological mechanisms, additional annotation based on the NCBI nr database was performed for all of the 2,135 unigenes with differential expression patterns. A total of 1,558 (72.97%) of the unigenes had unknown functions. The remaining 577 unigenes were divided into 37 groups (Additional file 12: Figure S8C), in which the transcription factors (57), protein kinases (44), photosynthesis proteins (40), transport (40), lipid biosynthesis proteins (39), ubiquitin system (35), transcription machinery (30) and glycan biosynthesis (26) occupied a large proportion (57.36% of the total known unigenes). After comparing these results with studies related to fruit [24,44-46], we observed both many similarities and specificities for flowers.

A reduction in light intensity is thought to be the most direct effect of shading. It has been hypothesized that photosynthetic rates are significantly altered by different levels of shading [47,48]. Zhu et al. [24] found that approximately 6% of shade-response genes are related to photosynthesis. In another study, approximately 2% of litchi (*Litchi chinensis*) unigenes induced by shading were found to be related to photosynthesis [26]. In this work, we found that 1.87% of chrysanthemum unigenes were related to photosynthesis (Additional file 13: Table S5), which is similar to the study of litchi [26]. Among them, 92.5% of the unigenes documented were repressed; these unigenes were mainly involved in photosystem and electron transport system, antenna proteins, chloroplastid, carbon fixation in photosynthetic organisms, and oxidative phosphorylation and nitrogen metabolism; the remaining up-regulated unigenes only included genes involved in chlorophyll biosynthesis.

Shading also had a large impact on protein ubiquitination and degradation. For example, F-box proteins, 26S proteasome subunit proteins, the ubiquitin E3 ligase complex including cullin and ubiquitin-conjugating enzymes were regulated in response to shading, which is in agreement with the findings in apples [24] and litchi [26]. Shading may also affect the expression of stress-response genes [26]. For instance, some unigenes involved in the cellular response to salt, drought and disease stress were induced after shading (Additional file 13: Table S5). It has been previously reported that shading has an effect on cell-wall loosening and degradation [24,45]. In our study, 14 cell-wall degradation-related genes were induced after shading. These genes included eight up-regulated unigenes mainly involved in pectin methylesterase, polygalacturonase-inhibiting protein, cellulose synthase, cell wall glycoprotein, wall-associated receptor kinase and extensin-like protein, and six down-regulated unigenes mainly involved in glycine-rich cell wall structural protein, cell wall-associated hydrolase, putative extensin and leucine-rich repeat extensin-like protein. These results indicated that ray florets activate cell-wall formation in response to low-light stress. Moreover, the generation of reactive oxygen species and differentially expressed genes related to cell wall decoration were also found in chrysanthemums (Additional file 13: Table S5).

Fruit and floral development are highly dependent on the carbohydrate supply. In response to shading, eight differentially expressed unigenes encoding enzymes involved in carbohydrate metabolism were identified (i.e., the metabolisms of ascorbate, starch, sucrose, fructose, mannose, galactose and gluconeogenesis); the majority of these unigenes were up-regulated (Additional file 13: Table S5). This result is in agreement with the findings in apples [24] and litchi [26]. The up-regulated expression of these genes is likely a direct or indirect early response of the ray florets to the carbohydrate storage.

Transcription factors, protein kinases and protein phosphatases are all related to the transcription process in plants. In the current study as many as 35 up-regulated and 22 down-regulated chrysanthemum unigenes under shading conditions were found to be related to transcription factors, mainly including MYB, bHLH, WD40, zinc finger protein, MADS-box, Homeobox, Ankyrin, MYC, RING finger, WRKY, NAC and GATA-factors. The differentially expressed unigenes related to protein kinases included six groups: CAMK, CK1, TKL, Atypical protein, Histidine protein kinases and other groups (such as receptor-like protein kinase, putative protein kinase, shikimate kinase and calmodulin-binding transcription activator). For protein phosphatases, both the up- and down-regulated unigenes possessed functions of phosphate and amino acid transporters (Additional file 13: Table S5).

Some research groups have reported that shading treatment affected the biosynthesis of many genetic materials and biological processes [11,23,26]. Unigenes related to DNA replication proteins and the chromosome or associated with the biogenesis of transfer RNA, mitochondria and the ribosome were found to be differentially expressed in the present study (Additional file 13: Table S5). A complex combination of signalling pathways has been proposed to play important roles in the modulation of fruit development. Hormone signalling is important for the coordination of fruit growth, fruit abscission and plant defence [49,50]. In our study, as many as 14 differentially expressed unigenes were hormone-related and involved in the signal transduction pathways of auxins, brassinosteroids, jasmonic acid, abscisic acid and indole. In contrast to the study of litchi [26], not all of these genes were up-regulated: two auxin-binding proteins, one putative auxin binding protein, one indole-3-acetic acid hydrolase and one jasmonic acid were down-regulated, indicating that the expression of these hormone-related genes are specific to flowers.

Beside the above mentioned functions, we also observed many differentially expressed unigenes related to transportation (transport and ion channels), floral development (developing and circadian rhythm), proteins (chaperones and folding catalysts, GTP-binding proteins, retrotransposon proteins, lectins, sweet proteins, glutathione metabolism and metallothionein) and other metabolisms (lipid biosynthesis proteins, glycan biosynthesis, gibberellin biosynthesis, amino acid related enzymes, anthocyanin biosynthesis, other secondary metabolites, tricarboxylic acid cycle, terpenoids and polyketides, cofactors and vitamins). Many of these results are similar to the studies on fruit, indicating that several homogeneous biological reactions in response to shading exist both in fruit and flowers. However, in the present study we observed that genes related to anthocyanin biosynthesis were repressed after shading, which has not been previously reported. The detailed analyses are shown below.

### Metabolic pathways responding to shading during capitulum development

To provide a global view of genes that are modulated in response to shading during chrysanthemum metabolism, the 923 and 1,212 up- and down-regulated unigenes were submitted for analysis via on-line iPath explorer version 2.0 (Additional file 14: Figure S9). iPath is an open-access online tool that integrates 123 KEGG maps from 183 species [51]. It has been widely used in association with RNA-Seq transcriptomics [52], for example, in the analysis of *Solanum glandular* trichomes [53]. The dynamic changes and magnitudes of absolute expression during capitulum development are shown in Additional file 14: Figure S9. The lines shown in this figure indicate genes modulated in response to shading that mapped to pathways, including the metabolism of lipids, carbohydrates, amino acids, nucleotides and energy metabolism. Blue, green and yellow lines indicate that the expression of most members in a specific gene family did not differ significantly, were down-regulated and were irregularly regulated during capitulum development, respectively. Importantly, some pathways (indicated by red lines) showed enhancement, such as pentose phosphate metabolism (Additional file 14: Figure S9B) that generates triphosphopyridine nucleotides to increase the metabolic rate during capitulum development; moreover, the expression of genes involved in the tricarboxylic acid cycle also increased Additional file 14: Figure S9B). In the present study, the iPath application provided an interactive metabolic network associated with gene expression changes during capitulum development in chrysanthemums. Interestingly, we found that almost all the structural genes participating in the anthocyanin metabolic pathway were up-regulated during capitulum development (Additional file 14: Figure S9B). This pathway represents the only complete metabolic pathways modulated in response to both light and floral development that we observed.

Light is a very important environmental factor that affects floral organ development and the metabolic pathway. Biosynthesis of phenolic compounds is known to be sensitive to light environments, which reflects the possible role of these compounds in photoprotection in plants [54]. Among these metabolic pathways, the molecular mechanism by which light affects anthocyanin biosynthesis has not been clearly revealed. Light also plays a key role in the development of floral organs in some plants. For example, after shading during the early-stage inflorescence of the Barberton daisy, Meng et al. [40] found that both the pigmentation of ray florets and the elongation of inflorescence were repressed. Further study has shown that light promoted the transverse and longitudinal development of cells in the Barberton daisy ray florets. In the present study, when the bud-stage capitulum of the chrysanthemum was shaded, the capitulum development was not repressed. This finding indicates that the influence of light on floral organ development may differ among species.

### Expression patterns of genes involved in the anthocyanin metabolic pathway

**Figure 4:**
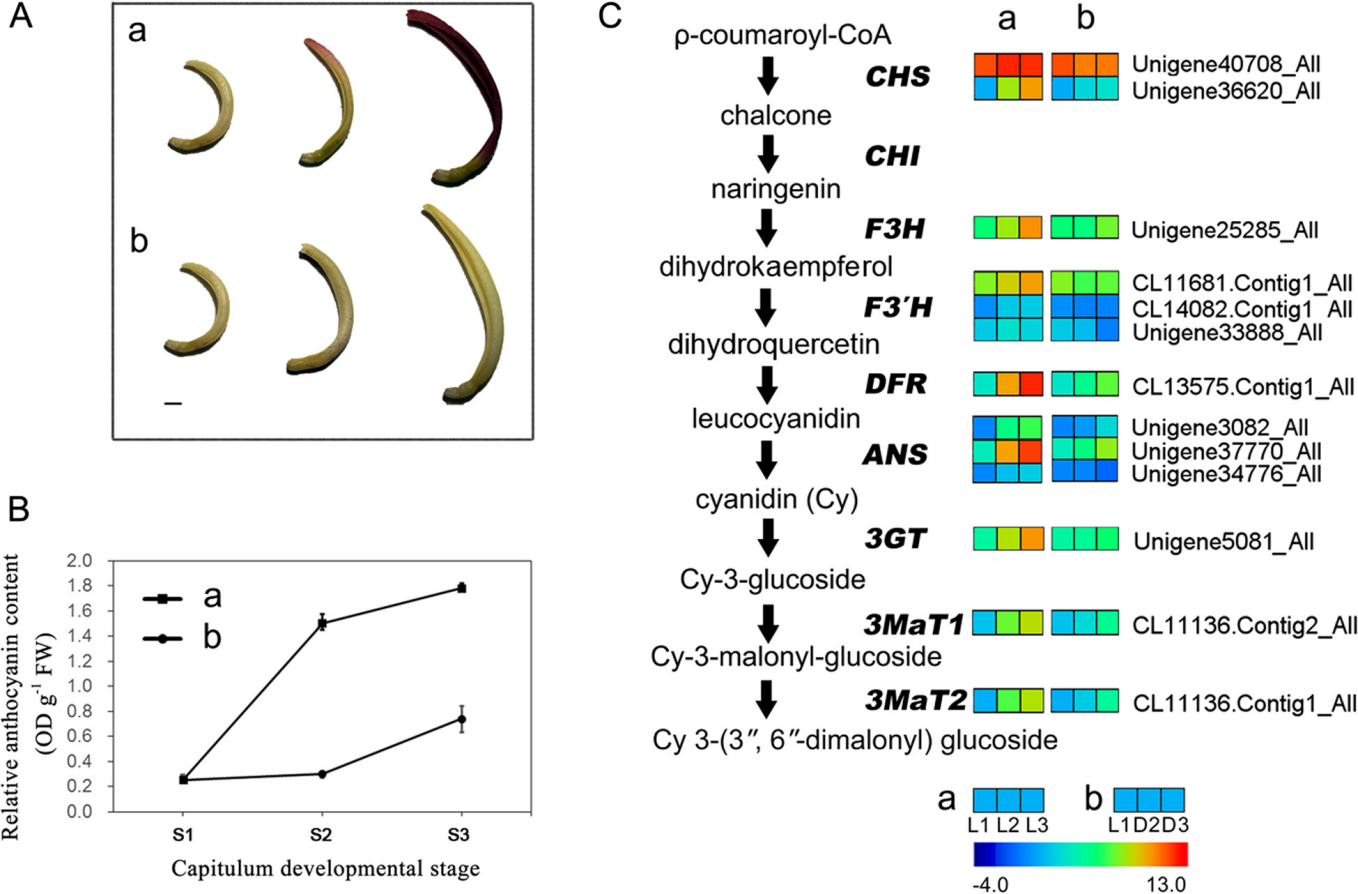
**Schematic of physiological and metabolic data related to chrysanthemum flower colour during capitulum development. A**, Changes in colour phenotypes of ray florets during capitulum development. Scale bar = 0.5 cm. **B**, Changes in relative anthocyanin content during the first three capitulum developmental stages (S1, S2 and S3) detected by HPLC analysis. The x-axis and y-axis represent the relative anthocyanin content and the three capitulum developmental stages, respectively. **C**, Anthocyanin biosynthesis pathway. Enzyme names, unigene names and expression patterns are indicated on the right of each step. The expression patterns of each unigene or contig are shown using different grids from blue (−4.0) to red (13.0), with the values representing the log_10_ FPKM values. The higher the grid values, the higher the gene expression levels. a and b represent the light and dark libraries, respectively.

During the first three stages (S1, S2 and S3) of capitulum development, the ray florets undergo a rapid change in flower colour under light conditions, ranging from white on the whole petal to pink on the tip of the petal to purple on the whole petal (Figure 4A). This change is due to the increase in relative anthocyanin content from 0.25 OD g^−1^ FW to 1.50 OD g^−1^ FW to 1.72 OD g^−1^ FW as detected by HPLC analysis (Figure 4B). In this work, unigenes participating in the anthocyanin metabolic pathway were selected and studied in detail. We observed that the expressions of 13 unigenes or contigs related to the eight genes encoding corresponding enzymes involved in the anthocyanin metabolic pathway were significantly up-regulated with the exception of *CHI* (Figure 4C).

In the present study, we found that ray floret colour deepened with capitulum development; accordingly, there was an increase in the relative anthocyanin content and the expression levels of key genes related to anthocyanin biosynthesis. The variation tendencies of the three were coincident. These findings are similar to studies on apple [55], anthura (*Anthurium andraeanum*) [56] and the Barberton daisy [40], indicating a relationship between floral development and the pigmentation of plants. This relationship may occur because the two processes might be regulated by the same upstream pathways, or colour formation might be strictly regulated by capitulum development.

The present study showed that the pigmentation of chrysanthemum ray florets was severely repressed after shading; the relative anthocyanin content decreased and the expression of almost all the structural genes was repressed. These findings indicated that anthocyanin biosynthesis in chrysanthemums relies on light. Therefore, light is the necessary induction factor for normal pigmentation in chrysanthemums during certain developmental stages and promotes anthocyanin biosynthesis mainly by activating the expression of genes related to anthocyanin biosynthesis.

The promoting effect of light on the transcriptional levels of structural and regulatory genes has been observed in many plants, such as *CHS* in arabidopsis [57-59], *DFR* and *MYBP1* in purple perilla (*Perilla frutescens*) [60], *PAL* in corn (*Zea mays*) [61], *CHS*, *F3H*, *DFR*, *LDOX* and *UFGT* in apple [14], *LAP1*, *MtMYBA* and *AN2* in alfalfa (*Medicago sativa*) [62,63], and *CHS*, *CHI*, *F3H*, *DFR*, *LDOX*, *UFGT* and *MYBA1* in grape [64]. Using transcriptomic analysis we obtained a comprehensive understanding of the changes in structural genes related to anthocyanin synthesis after shading at the transcriptomic level due to the elimination of the functional effects of different gene family members. In the present study, we found that the expression of most of the structural genes involved in the anthocyanin biosynthetic pathway was affected by light (with the exception of *CHI*), indicating that the light-induced biosynthetic mechanism of anthocyanin in chrysanthemums is species-specific.

Gene expression decreased 2- to 36-fold under shading conditions, with high levels of repression of the expression of anthocyanin-specific branch genes *DFR*, *ANS*, *3GT*, *3MT1* and *3MT2*. The expression of unigene 34776_All (*ANS*) after shading was repressed almost completely; thus, this gene was inferred to be a key structural gene related to the effect of light on anthocyanin biosynthesis.

In conclusion, the regulation of anthocyanin biosynthesis in chrysanthemums by light is in turn regulated by the developmental stage of the capitulum; in other words, the pigmentation process in chrysanthemums is simultaneously regulated by light and capitulum development.

### Differential effects on transcription factors regulating anthocyanin biosynthesis between the light and dark libraries

**Figure 5:**
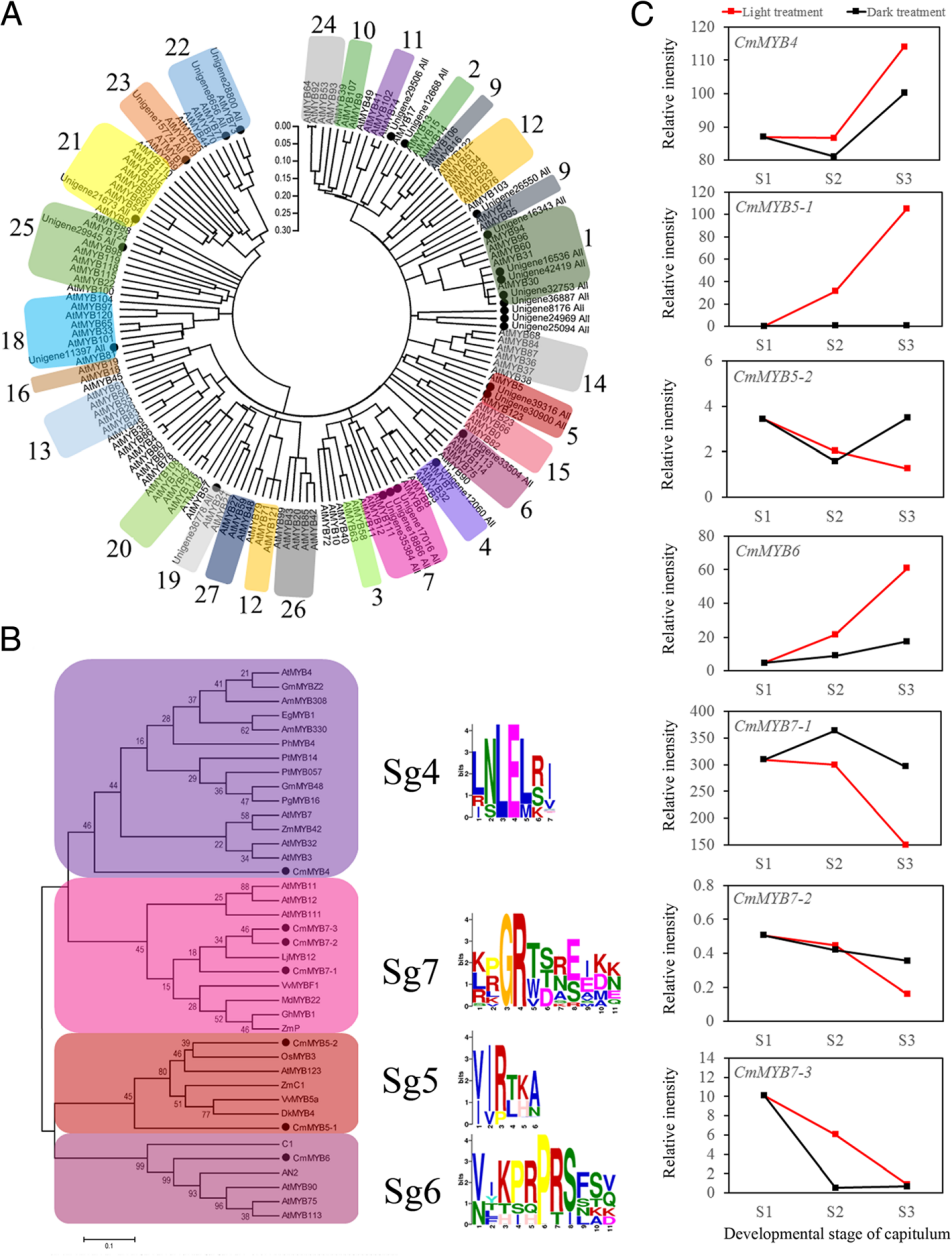
**Evolutionary relationships of R2R3-MYBs and validation of CmMYBs. A**, Evolutionary relationships of 125 AtMYBs and 34 CmMYBs. Full-length amino acid sequences of R2R3-MYBs from the chrysanthemum transcriptome dataset and arabidopsis genome were first aligned using ClustalW in MEGA5. The phylogenetic tree was constructed according to the neighbour-joining method. Branches corresponding to partitions reproduced in less than 50% of the bootstrap replicates were collapsed. The evolutionary distances were computed using the *p*-distance method. All ambiguous positions were removed for each sequence pair. Evolutionary analyses were conducted in MEGA5. The black circle represents 34 CmMYBs. **B**, Phylogenetic tree of R2R3-MYBs and conserved motifs of chrysanthemum and other plants in subgroups 4, 5, 6 and 7. The black circle represents CmMYBs. Conserved motifs detected by MEME are listed behind each subgroup. **C**, Validation of CmMYBs in subgroups 4, 5, 6 and 7. The x-axis and y-axis represent the three developmental stages of the capitulum and the FPKM values, respectively. Red and black lines indicate the light and shading treatments, respectively.

A total of 34 chrysanthemum R2R3-MYBs and 125 arabidopsis R2R3-MYBs were used to construct a phylogenetic tree (Figure 5A). CmMYBs were integrated with AtMYBs in clustered phylogenetic clades or subclades and divided into 25 MYB subgroups overall (Figure 5A). The resulting tree exhibited similar basic subgroups to those observed by Matus et al. [65] with the exception of Sg8 and Sg17. MYBs in Sg4, Sg5, Sg6, Sg7 and Sg15 were reported to be regulators of anthocyanin and proanthocyanidin biosynthesis [66]. In the present study, there was one CmMYB in Sg4 (unigene 12060, renamed as *CmMYB4*), two in Sg5 (unigene 30900 and unigene 39316, renamed as *CmMYB5-1* and *CmMYB5-2*, respectively), one in Sg6 (unigene 33504, renamed as *CmMYB6*), and three in Sg7 (unigene 17016, unigene 18866 and unigene 35384, renamed as *CmMYB7-1*, *CmMYB7-2* and *CmMYB7-3*, respectively); no CmMYBs were found in Sg15.

According to Matus et al. [65], most of MYBs belonging to the same subgroup share motifs outside of the MYB domain. Consistent motifs were generally observed among arabidopsis, chrysanthemum and many other ornamental plants using MEME (Figure 5B), including Sg4 (LNLEL[R/S]I) [67], Sg5 ([V/I]IR[T/L][K/H]A) [68], Sg6 ([V/N]IKPRPRSFS[Q/V]) [69], and Sg7 ([K/L/R][P/R]GR[T/W][S/D/T][R/N][E/S][I/E][K/D][K/N]) [70], further confirming the validity of the proposed tree (Figure 5A).

To investigate the expression patterns of these seven CmMYBs during capitulum developmental processes under light and shading conditions, the relative intensities (FPKM value) of these genes were further analysed (Figure 5C). The results showed that the expression of *CmMYB4*, *CmMYB5-1* and *CmMYB6* increased under both of the light conditions in agreement with capitulum development and were repressed after shading; in contrast, the opposite trend was observed for *CmMYB7-1*. Therefore, we inferred that *CmMYB4*, *CmMYB5-1* and *CmMYB6* might be regulation enhancers of light-induced anthocyanin biosynthesis in chrysanthemums, while *CmMYB7-1* might participate in the negative regulation of anthocyanin biosynthesis.

**Figure 6:**
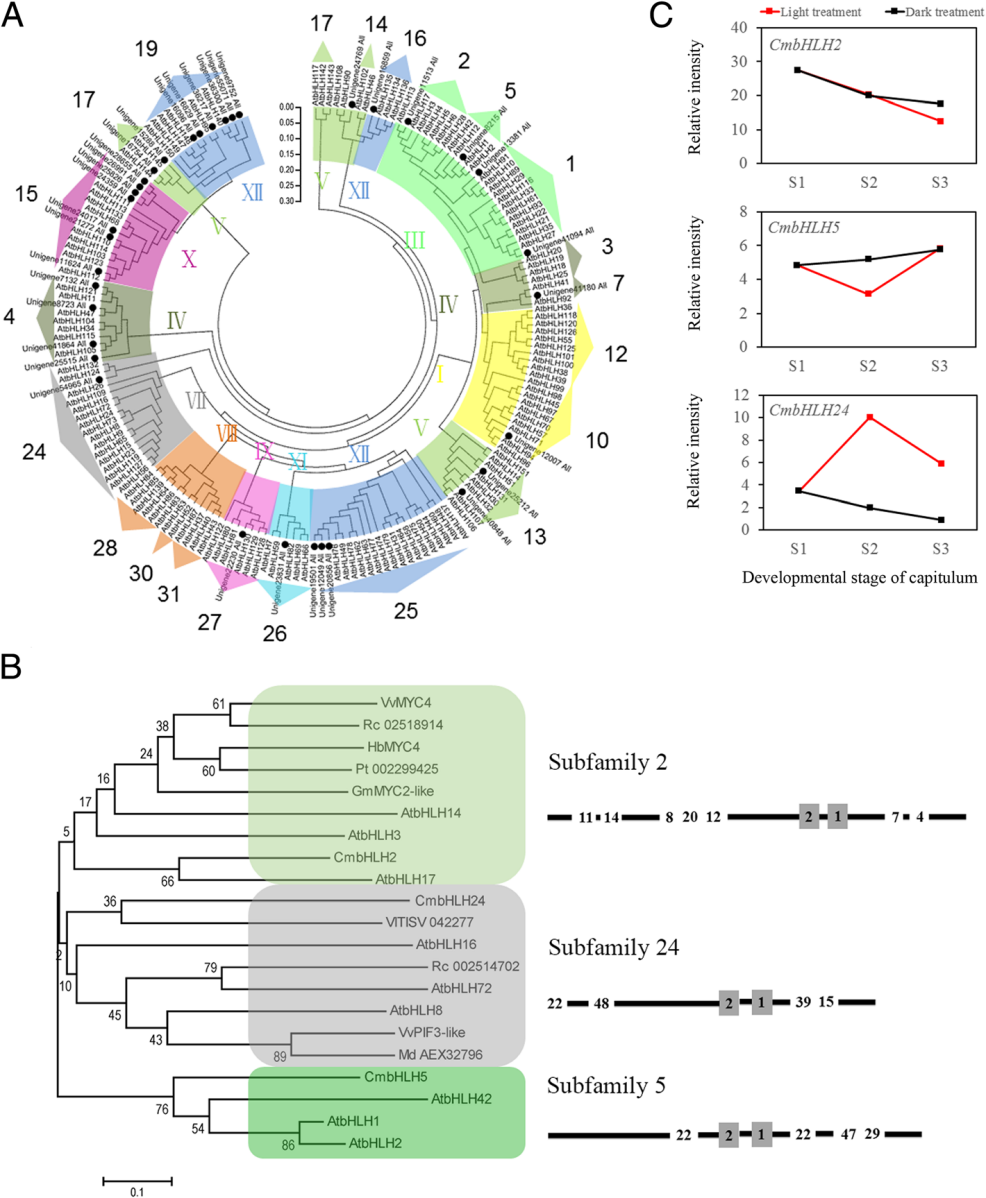
**Evolutionary relationships of bHLHs and validation of CmbHLHs. A**, Evolutionary relationships of 149 AtbHLHs and 25 CmbHLHs. Full-length amino acid sequences of bHLHs in the chrysanthemum transcriptome dataset and arabidopsis genome were first aligned using ClustalW in MEGA5. The phylogenetic tree was constructed according to the neighbour-joining method. Branches corresponding to partitions reproduced in less than 50% of bootstrap replicates were collapsed. The evolutionary distances were computed using the *p*-distance method. All ambiguous positions were removed for each sequence pair. Evolutionary analyses were conducted in MEGA5. The black circle represents 25 CmbHLHs. **B**, Phylogenetic tree of bHLHs and conserved motifs of chrysanthemum and other plants in subfamilies 2, 5 and 24. The black circle represents CmbHLHs. Conserved motifs are listed behind each subfamily. **C**, Validation of CmbHLHs in subfamilies 2, 5 and 24. The x-axis and y-axis represent the three developmental stages of the capitulum and the FPKM values, respectively. Red and black lines indicate the light and dark treatments, respectively.

Based on conserved short motifs identified outside of the DNA binding domain, Heim et al. [71] classified 113 observed bHLH proteins into 12 groups numbered from I to XII. In this system, members of IIId, IIIe, and IIIf function as transcription factors regulating genes involved in flavonoid metabolism [71]. Carretero-Paulet et al. [72] presented a comprehensive classification as well as a structural and evolutionary analysis of the *bHLH* gene family in plants, classifying 638 observed *bHLH* genes from arabidopsis, poplar (*Populus*), rice (*Oryza sativa*), mosses, and algae species into 32 subfamilies. Notably, subfamilies 2, 5 and 24 were identified as regulators of flavonoid or anthocyanin metabolism. Using the same methods, 25 CmbHLHs and 149 AtbHLHs were constructed into a phylogenetic tree and divided into 10 groups (inside number) and 21 subfamilies (outside number; Figure 6A), excluding the subfamilies 6, 8, 9, 11, 18, 20, 21, 22, 23, 29 and 32. The number of CmbHLH associations with flavonoid or anthocyanin biosynthesis was less than expected, with one CmbHLH identified in subfamily 2 (unigene 11513, renamed as *CmbHLH2*), subfamily 5 (unigene 8215, renamed as *CmbHLH5*) and subfamily 24 (unigene 8723, renamed as *CmbHLH24*), respectively.

The HLH domain commonly provides information on specific DNA-binding abilities due to the amphipathic affinity of its N-terminal. This is especially apparent in the critical His-Glu-Arg (H-E-R) motif located at positions 5, 9, and 13. These HER sites have been shown to bind to a variation of the E-box hexanucleotide sequence (E-box: 5’-CANNTG-3’, variation G-box: 5’-CACGTG-3’) [73]. The HLH region is composed of two hydrophobic α-helices linked by a divergent loop and is marked by the alignment of amino acids of subfamilies 2 and 24 in Figure six (A). The activation or repression domains located outside of the DNA binding domain are important for the regulation of gene expression. These sequences usually contain short motifs that are conserved between related proteins in different species. In most cases, the protein architecture is remarkably conserved within specific subfamilies, providing further support for phylogenetic analysis based on bHLH domains.

Based on 50 motifs of variable lengths (8–80 amino acids) summarized from the 638 bHLHs examined by Carretero-Paulet et al. [72], conserved motif architecture structures in subfamilies 2, 5 and 24 were 11-14-8-20-12-2-1-7-4, 22-2-1-22-47-29 and 22-48-2-1-39-15, respectively (Figure 6B). Similar to CmMYBs, we analysed the relative intensity of these three *CmbHLH* genes (Figure 6C). The results showed that only *CmbHLH24* revealed a clear expression pattern and was repressed after shading. Therefore, we inferred that *CmbHLH24* might be a regulation enhancer of light-induced anthocyanin biosynthesis in chrysanthemums.

In general, the expression of transcription factors is induced by external signals and affects the pigmentation of plant fruits, leaves and flowers through their influence on structural gene expression levels related to anthocyanin biosynthesis [74]. For example, in the study of petunia, the authors found that transcript PhMYB27 (a putative S1S2-MYB active repressor) was highly expressed during non-inductive shade conditions and repressed during high light. The competitive inhibitor PhMYBx (S2-MYB) was expressed under high light, which suggested that it may provide feedback repression [75]. Correlations have been found between light, anthocyanin biosynthesis and transcript levels of MYB. It has been suggested that MYB determined the light-induced phenotype in petunias [4] and played an important role in the regulation of anthocyanin biosynthesis in corn in response to different light qualities [76,77]. In fruit, the enhancement of MYB expression by sunlight was also observed during fruit pigmentation. In pear (*Pyrus* spp.) fruit under normal sunlight conditions for eight days after bag removal, PyMYB10 transcripts were approximately 12-fold higher than those observed in the bagged fruit [78]. When dark-grown apple fruit were exposed to sunlight, MdMYB1 transcript levels increased over several days [14]. In studies investigating the mechanism, promoter structure differentiation of MYB transcription factor RLC1 was shown to induce red leaf pigmentation in empire red leaf cotton under light conditions [7]. All of the above reports indicated the significance of transcription factors on the regulation of the anthocyanin biosynthetic process in higher plants. In the present study, we identified five important transcription factors that might be key genes regulating anthocyanin biosynthesis and correspondingly resulting in the change of flower colour phenotypes in chrysanthemums. These findings are similar to many studies related to the anthocyanin-light relationship in other multiplication organs. However, the elucidation of their functions requires more elaborate studies.

### Validation of the expression of genes related to anthocyanin biosynthesis in chrysanthemum

**Figure 7:**
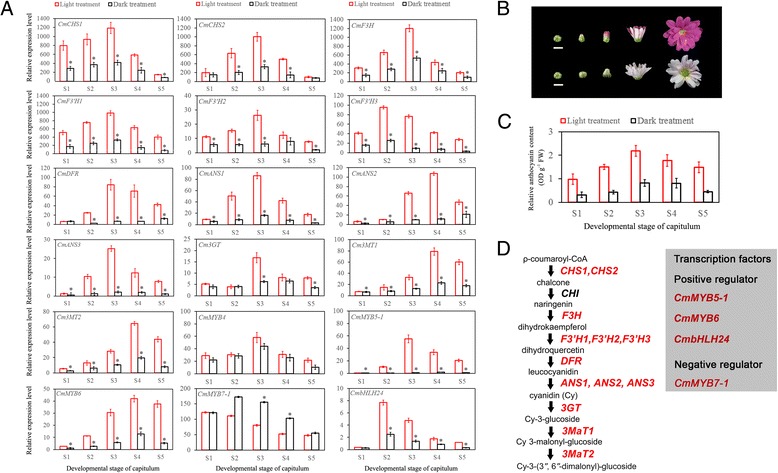
**Validation of the expression of genes related to anthocyanin biosynthesis in chrysanthemums. A**, Relative expression levels of 13 functional genes related to anthocyanin biosynthesis and five transcription factors under different treatment conditions by RT-qPCR. The x-axis and y-axis represent the five developmental stages of the capitulum and the relative expression level of each gene, respectively. Red and black squares indicate light and shading treatments, respectively. *represents significance at the 0.05 level. All gene expression levels were normalized against *CmPP2A*. **B**, The phenotype of the capitulum during five developmental stages. The upper part depicts the capitulum treated under light condition, while the lower part depicts the capitulum treated under dark conditions. Scale bar = 1cm. **C**, The relative anthocyanin content of different samples detected by the HPLC method. The x-axis and y-axis represent the five developmental stages of the capitulum and the relative anthocyanin content, respectively. Red and black squires indicate light and shading treatment, respectively. **D**, The inferred light-induced anthocyanin biosynthesis pathway in chrysanthemums.

To verify the reliability of the chrysanthemum transcription data, all five transcription factors (*CmMYB4*, *CmMYB5-1*, *CmMYB6*, *CmMYB7-1* and *CmbHLH24*) and 13 structural genes involved in the anthocyanin biosynthetic pathway (*CmCHS1*, *CmCHS2*, *CmF3H*, *CmF3’H1*, *CmF3’H2*, *CmF3’H3*, *CmDFR*, *CmANS1*, *CmANS2*, *CmANS3*, *Cm3GT*, *Cm3MT1* and *Cm3MT2*) in chrysanthemums were amplified from samples collected during the five capitulum developmental stages and two light conditions by RT-qPCR (Figure 7A). *PP2A* was used as a reference gene [79]. The results showed that all 13 structural genes showed the same expression patterns predicted by the transcription results: with the capitulum development, their expression first increased and then decreased. Moreover, significant differences between the light and shading samples were detected at the 0.05 level. The results were the same for the transcription factors. However, it is worth noting that although the expression of *CmMYB4* showed the same expression pattern as the 13 structural genes, there were no significant differences between the light and shading samples collected from the five capitulum developmental stages. Therefore, *CmMYB4* was eliminated from our results (Figure 7A). These results were consistent with the fading of flower colour phenotypes (Figure 7B) and changes in relative anthocyanin content (Figure 7C) under the same experimental conditions, indicating the high reliability of the chrysanthemum transcription data.

Based on the above results, a light-induced anthocyanin biosynthesis pathway in chrysanthemums was inferred (Figure 7D). Briefly, following the regulation of CHS1 and CHS2 in chrysanthemum ray florets, chalcone is generated from ρ-coumaroyl-CoA, which is further transferred to naringenin under the regulation of CHI. Next, F3H plays a key role in the generation of dihydrokaempferol. Then, under the regulation of F3’H1, F3’H2 and F3’H3, dihydrokaempferol is transferred to dihydroquercetin, and leucocyanidin is further generated due to the existence of DFR. Cyanidin, a substance unique to the metabolic pathway of anthocyanin in chrysanthemums, is generated from leucocyanidin under the regulation of enzymes ANS1, ANS2 and ANS3. Finally, cyanidin-3-*O*-(6″-*O*-malonyl-glucoside) and cyanidin-3-*O*-(3″,6″-*O*-dimalonyl-glucoside) are generated in order after the regulation of 3GT, 3MT1 and 3MT2, respectively. The whole metabolic pathway of the anthocyanin transcription factors, including three positive regulators (*CmMYB5-1*, *CmMYB6* and *CmbHLH24*) and one negative regulator (*CmMYB7-1*), play roles in the regulation of structural gene expression, although their functions and interactions with each other need further study.

## Conclusions

The pigmentation of the ray florets of chrysanthemum cultivar ‘Purple Reagan’ is dependent on light. During the light-induced pigmentation process, the expression of structural genes *CHS*, *F3H*, *F3’H*, *DFR*, *ANS*, *3GT* and *3MT* in the anthocyanin biosynthetic pathway (regulated by transcription factors *CmMYB5-1*, *CmMYB6*, *CmbHLH24* and *CmMYB7-1* in response to light) are the main contributors to the pigmentation of chrysanthemums.

### Availability of supporting data

The data sets supporting the results of this article are available in the LabArchives repository, https://mynotebook.labarchives.com/share/2554620161543841/MTMuMHw4MzgwMC8xMC9UcmVlTm9kZS8zNzEyMDY5NDg2fDMzcLjA=.

## Additional files

Additional file 1: Figure S1. The five samples used in the transcriptomic analysis. Scale bar = 0.5 cm. Additional file 2: Table S1. Primers used for RT-qPCR analysis. Additional file 3: Table S2. Summary of the chrysanthemum ray floret transcriptome. Additional file 4: Table S3. Length and gap distribution of contigs and unigenes from each library of *Chrysanthemum* ×*morifolium* ‘Purple Reagan’. Additional file 5: Figure S2. The distribution of unigene numbers and lengths. Additional file 6: Figure S3. Characteristics of the homology search of chrysanthemum unigenes. A, Venn diagram of the number of unigenes annotated by BLASTx with an E-value threshold of 10^−5^ against four protein databases. B, E-value distribution of the top BLASTx hits against the nr database for each unigene. C, Numbers and percentages of unigenes matching the 20 top species using BLASTx in the nr database. Additional file 7: Table S4. KEGG pathway annotation of chrysanthemum unigenes. Additional file 8: Figure S4. GO classification of chrysanthemum unigenes. Additional file 9: Figure S5. COG classification of chrysanthemum unigenes. Additional file 10: Figure S6. Overall expression profiles for the unigenes expressed in ray floret libraries during capitulum development. A, Light library. B, Dark library. Additional file 11: Figure S7. Differentially expressed unigenes between the light and dark libraries. A, Number of differentially expressed unigenes between samples L2 (under light condition) and D2 (under dark condition). B, Number of differentially expressed unigenes between samples L3 (under light condition) and D3 (under dark condition). C, Intersection between samples L2 versus D2 and L3 versus D3. The plus symbol, minus symbol and triangular symbol represent up-regulated, down-regulated and invariable unigenes, respectively. Additional file 12: Figure S8. Comparisons between up- and down-regulated unigenes classified in the GO, KEGG and nr databases. A, Functional classification of differentially expressed unigenes based on GO categorization. B, Pathway assignment based on the KEGG classification metabolism categories. C, Comparison of numbers between up- and down-regulated unigenes in nr analysis. a and b represent the up- and down-regulated unigenes after shading, respectively. Additional file 13: Table S5. Summary of differentially expressed unigenes annotated in the nr database. Additional file 14: Figure S9. Interactive pathway analysis of anthocyanin biosynthesis during capitulum development. A, Up-regulated genes after shading. B, Down-regulated genes after shading.
