# Supplementary figures and images for: Transcriptomic analyses reveal species-specific light-induced anthocyanin biosynthesis in chrysanthemum
Source: BMC Genomics. 2015 Mar 17;16(1):202. doi: 10.1186/s12864-015-1428-1 (PMC4404602; doi:10.1186/s12864-015-1428-1)

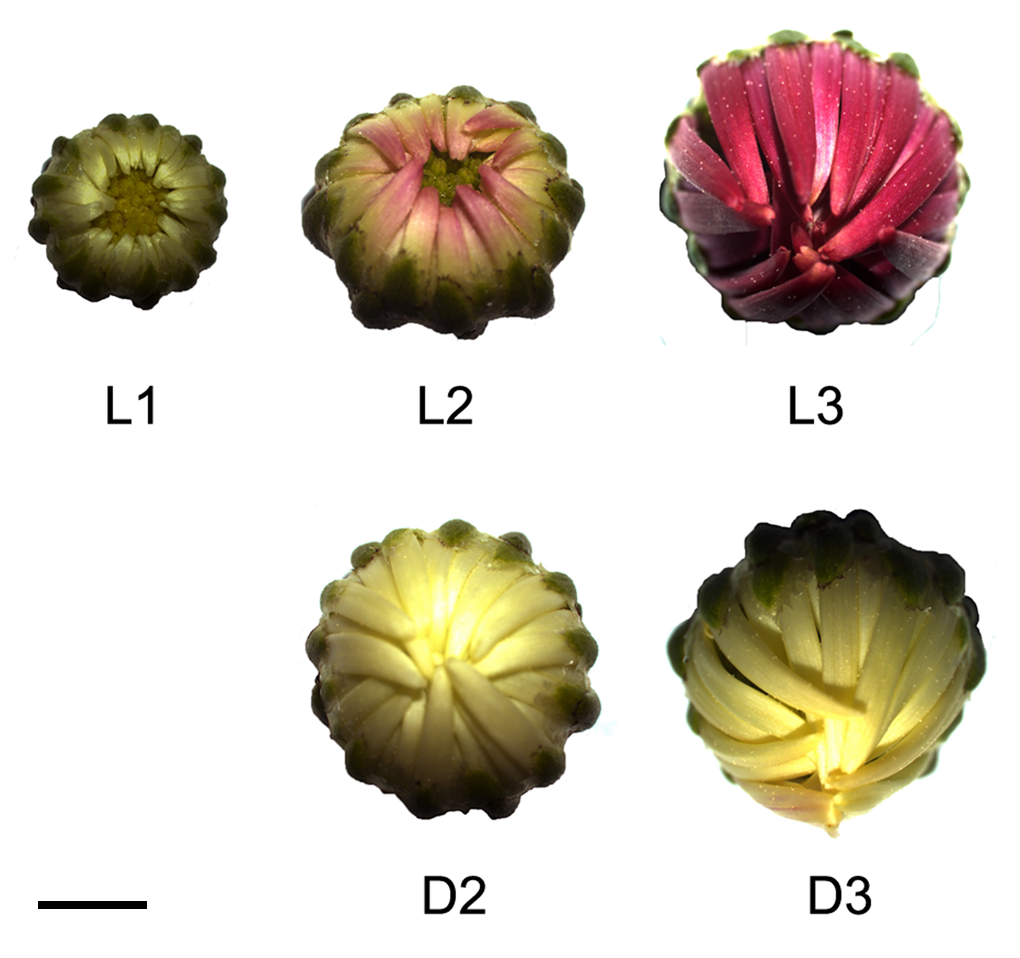

Supplement: Additional file 1: Figure S1. — The five samples used in the transcriptomic analysis. Scale bar = 0.5 cm. [file 12864_2015_1428_MOESM1_ESM.tiff]

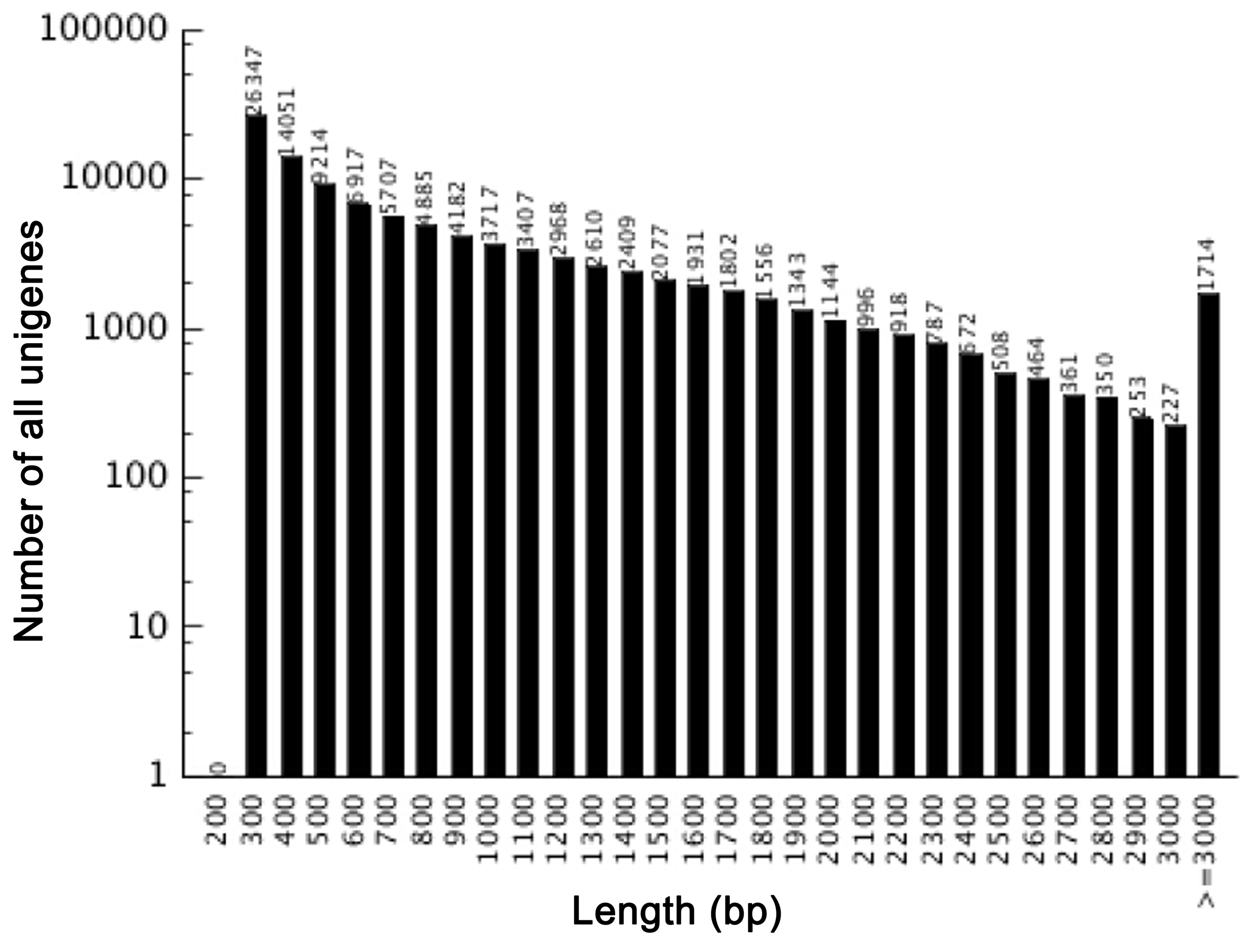

Supplement: Additional file 5: Figure S2. — The distribution of unigene numbers and lengths. [file 12864_2015_1428_MOESM5_ESM.tiff]

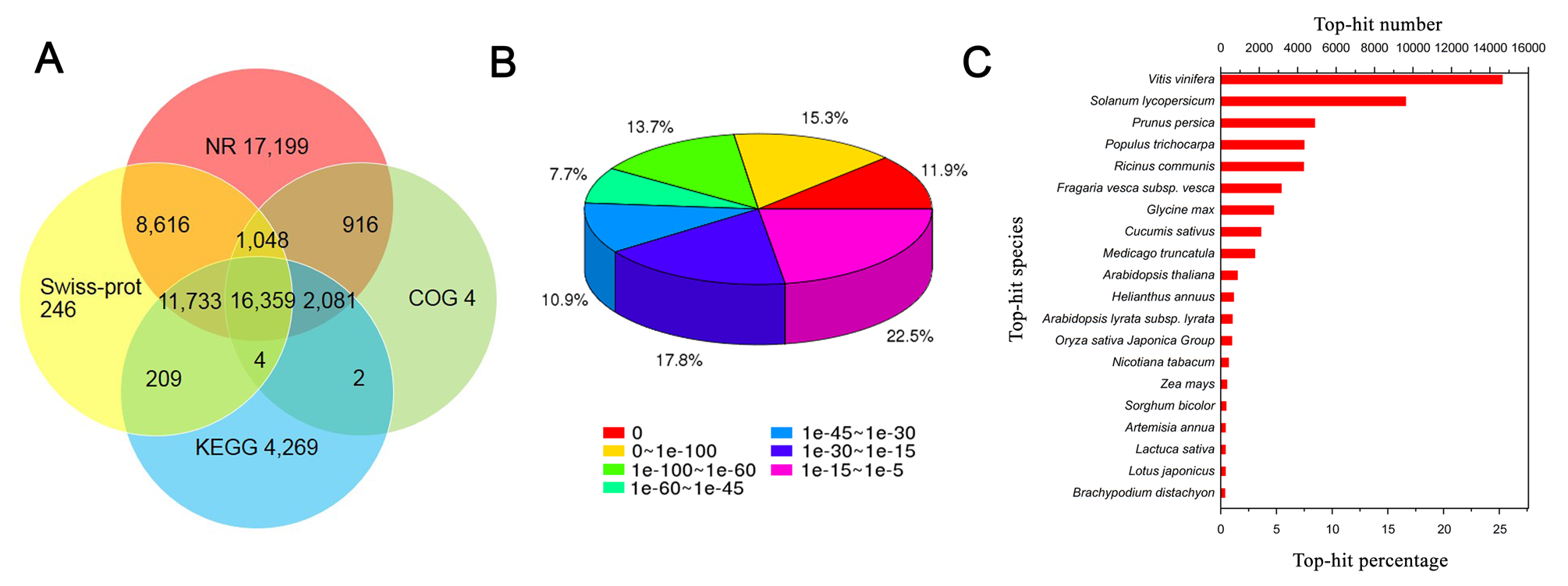

Supplement: Additional file 6: Figure S3. — Characteristics of the homology search of chrysanthemum unigenes. A, Venn diagram of the number of unigenes annotated by BLASTx with an E-value threshold of 10−5 against four protein databases. B, E-value distribution of the top BLASTx hits against the nr database for each unigene. C, Numbers and percentages of unigenes matching the 20 top species using BLASTx in the nr database. [file 12864_2015_1428_MOESM6_ESM.tiff]

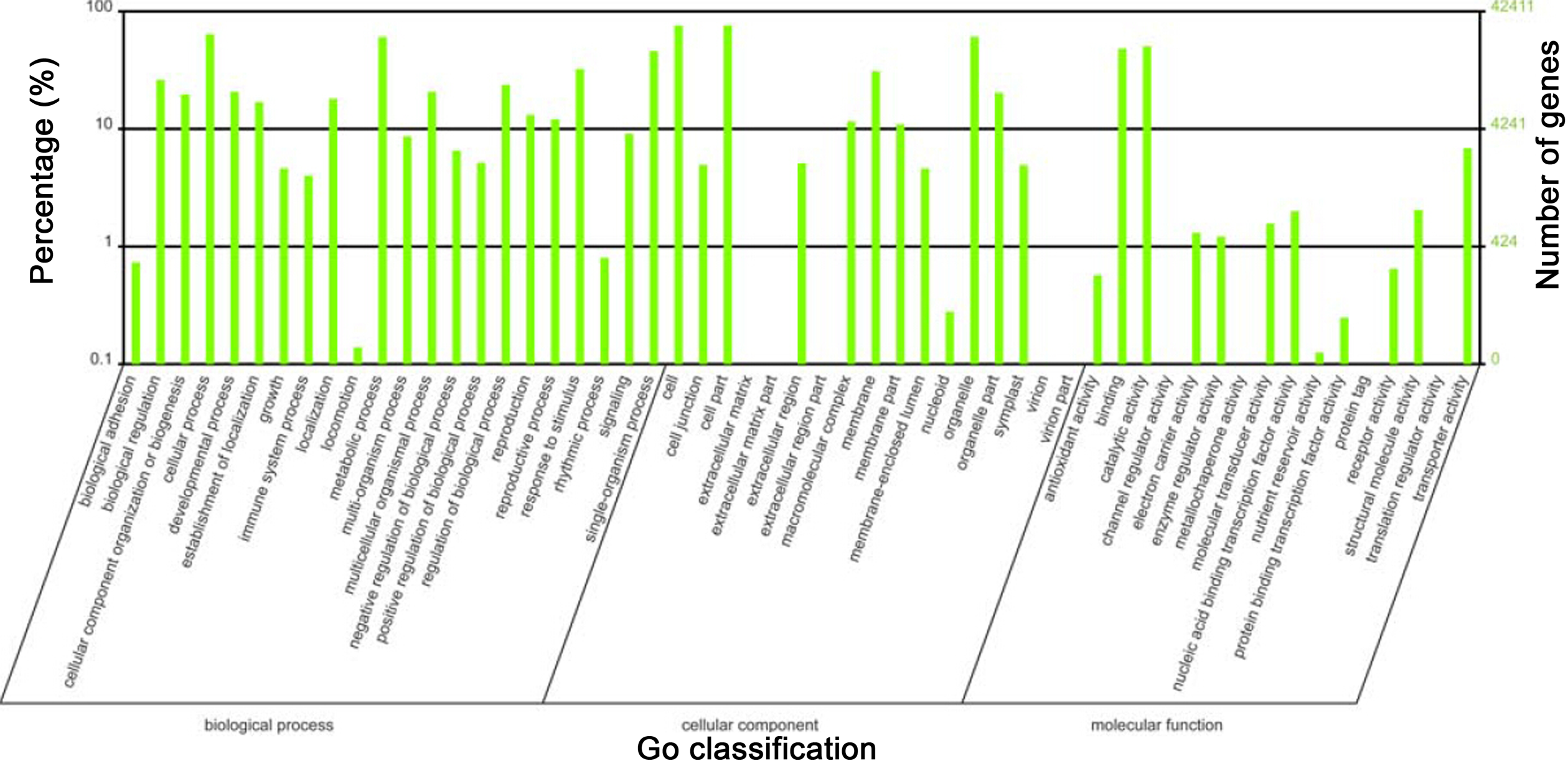

Supplement: Additional file 8: Figure S4. — GO classification of chrysanthemum unigenes. [file 12864_2015_1428_MOESM8_ESM.tiff]

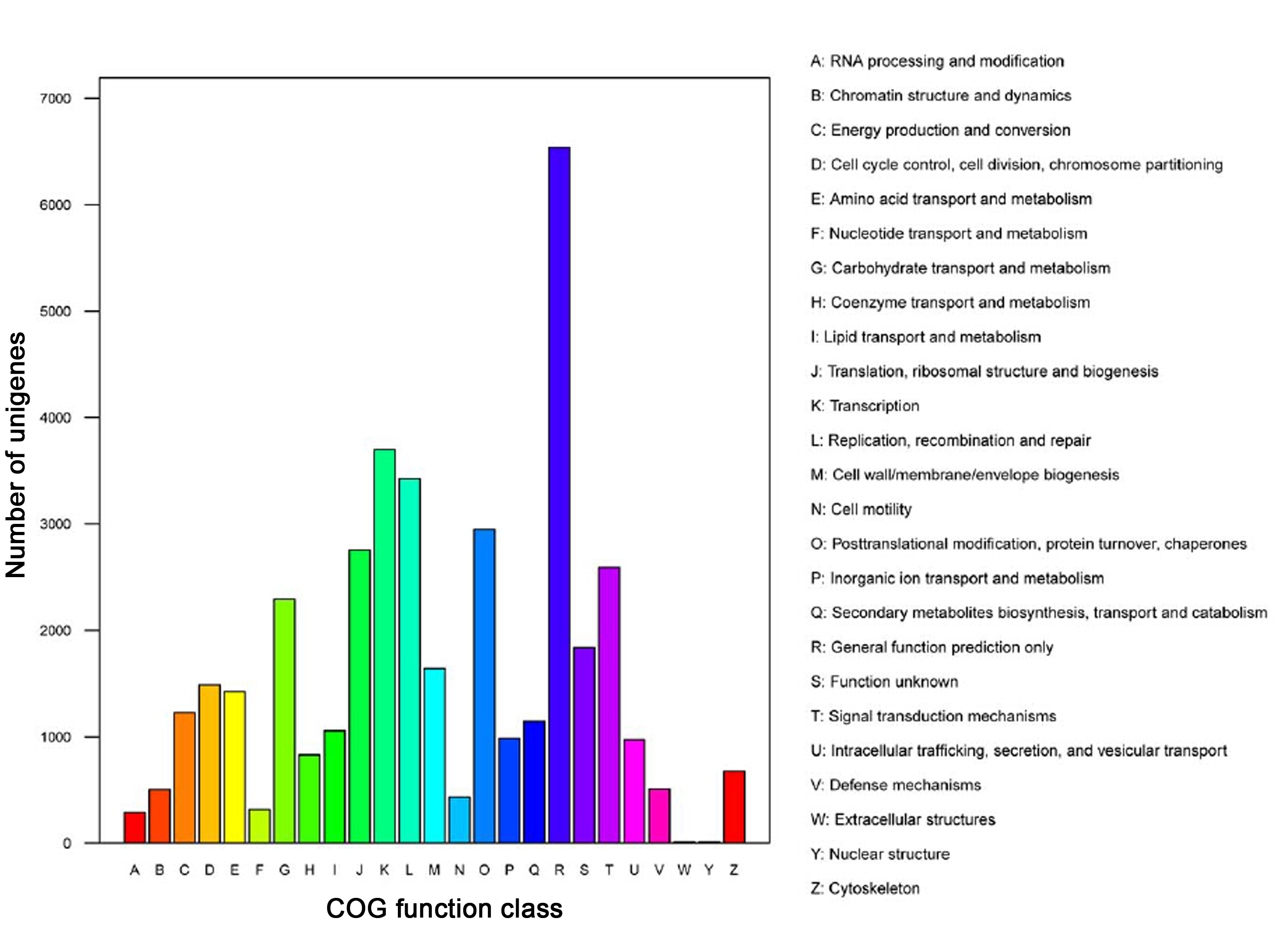

Supplement: Additional file 9: Figure S5. — COG classification of chrysanthemum unigenes. [file 12864_2015_1428_MOESM9_ESM.tiff]

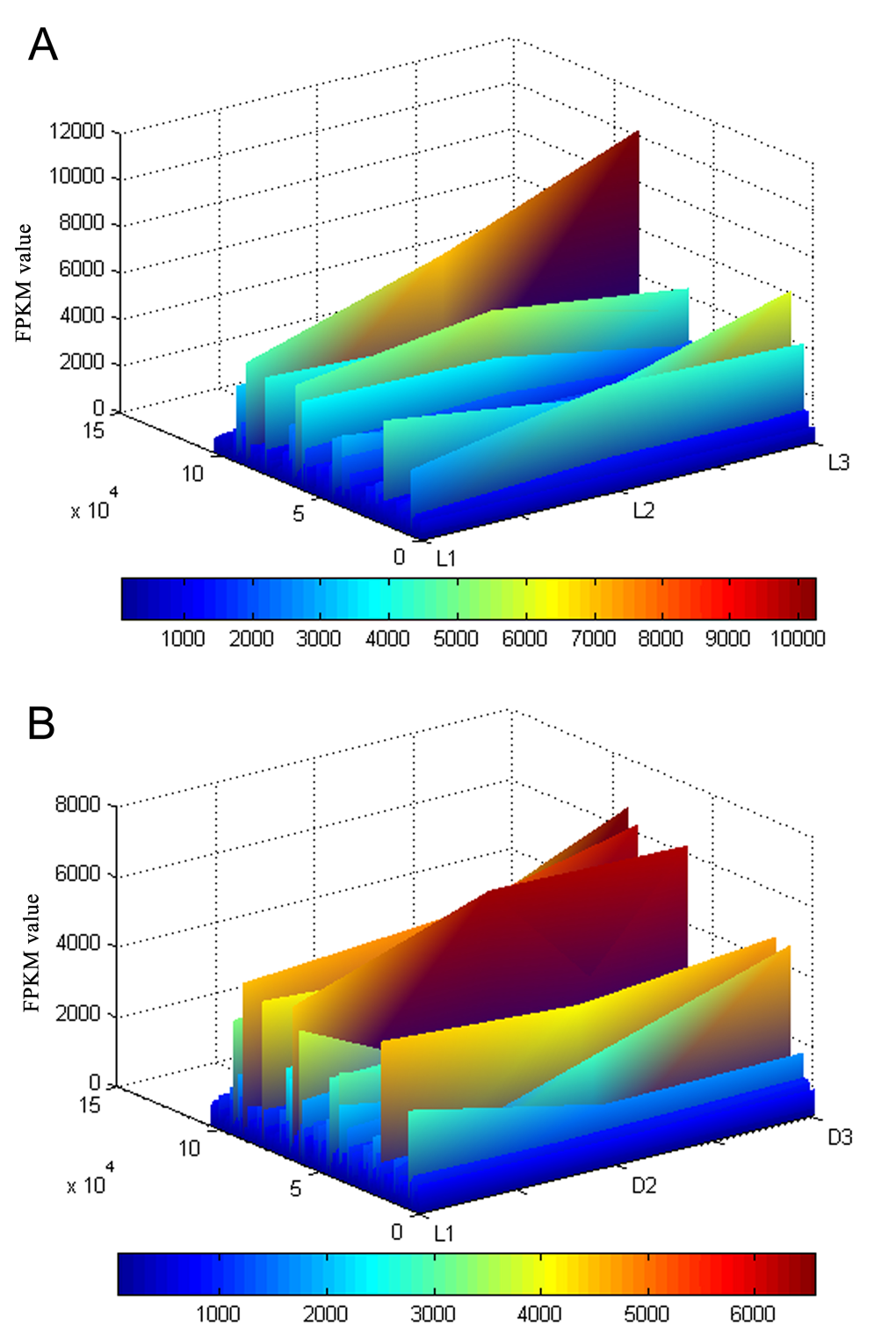

Supplement: Additional file 10: Figure S6. — Overall expression profiles for the unigenes expressed in ray floret libraries during capitulum development. A, Light library. B, Dark library. [file 12864_2015_1428_MOESM10_ESM.tiff]

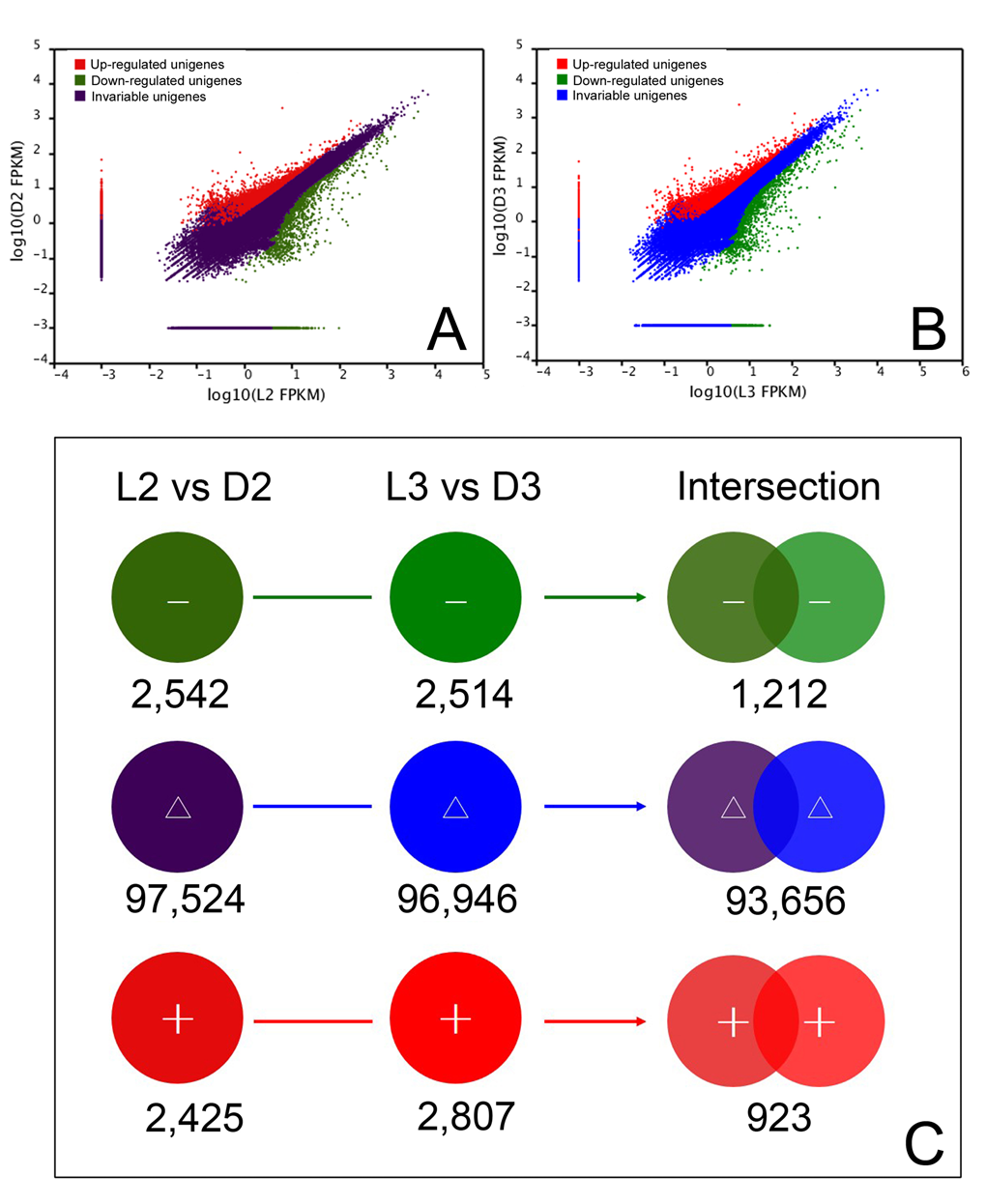

Supplement: Additional file 11: Figure S7. — Differentially expressed unigenes between the light and dark libraries. A, Number of differentially expressed unigenes between samples L2 (under light condition) and D2 (under dark condition). B, Number of differentially expressed unigenes between samples L3 (under light condition) and D3 (under dark condition). C, Intersection between samples L2 versus D2 and L3 versus D3. The plus symbol, minus symbol and triangular symbol represent up-regulated, down-regulated and invariable unigenes, respectively. [file 12864_2015_1428_MOESM11_ESM.tiff]

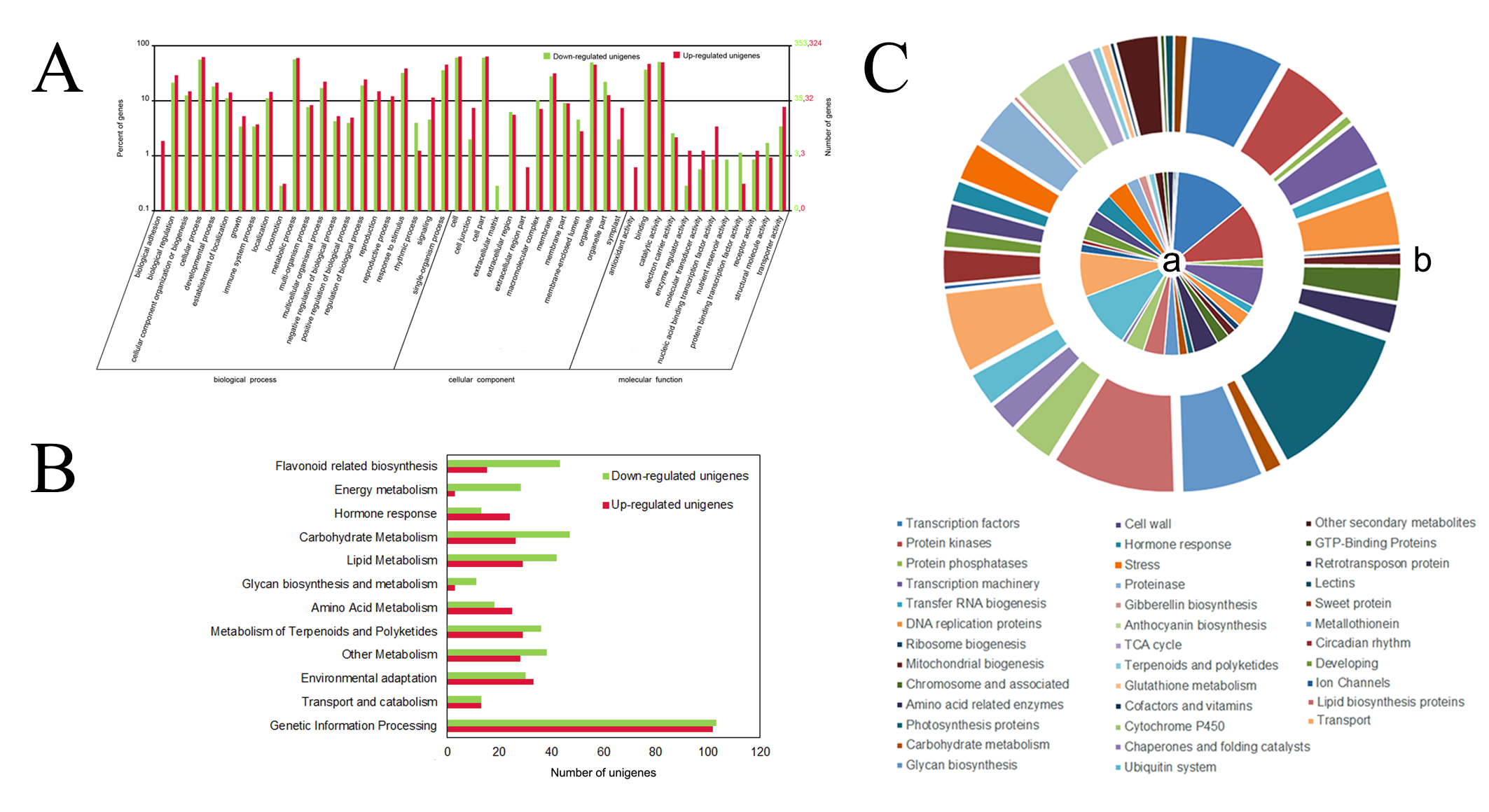

Supplement: Additional file 12: Figure S8. — Comparisons between up- and down-regulated unigenes classified in the GO, KEGG and nr databases. A, Functional classification of differentially expressed unigenes based on GO categorization. B, Pathway assignment based on the KEGG classification metabolism categories. C, Comparison of numbers between up- and down-regulated unigenes in nr analysis. a and b represent the up- and down-regulated unigenes after shading, respectively. [file 12864_2015_1428_MOESM12_ESM.tiff]

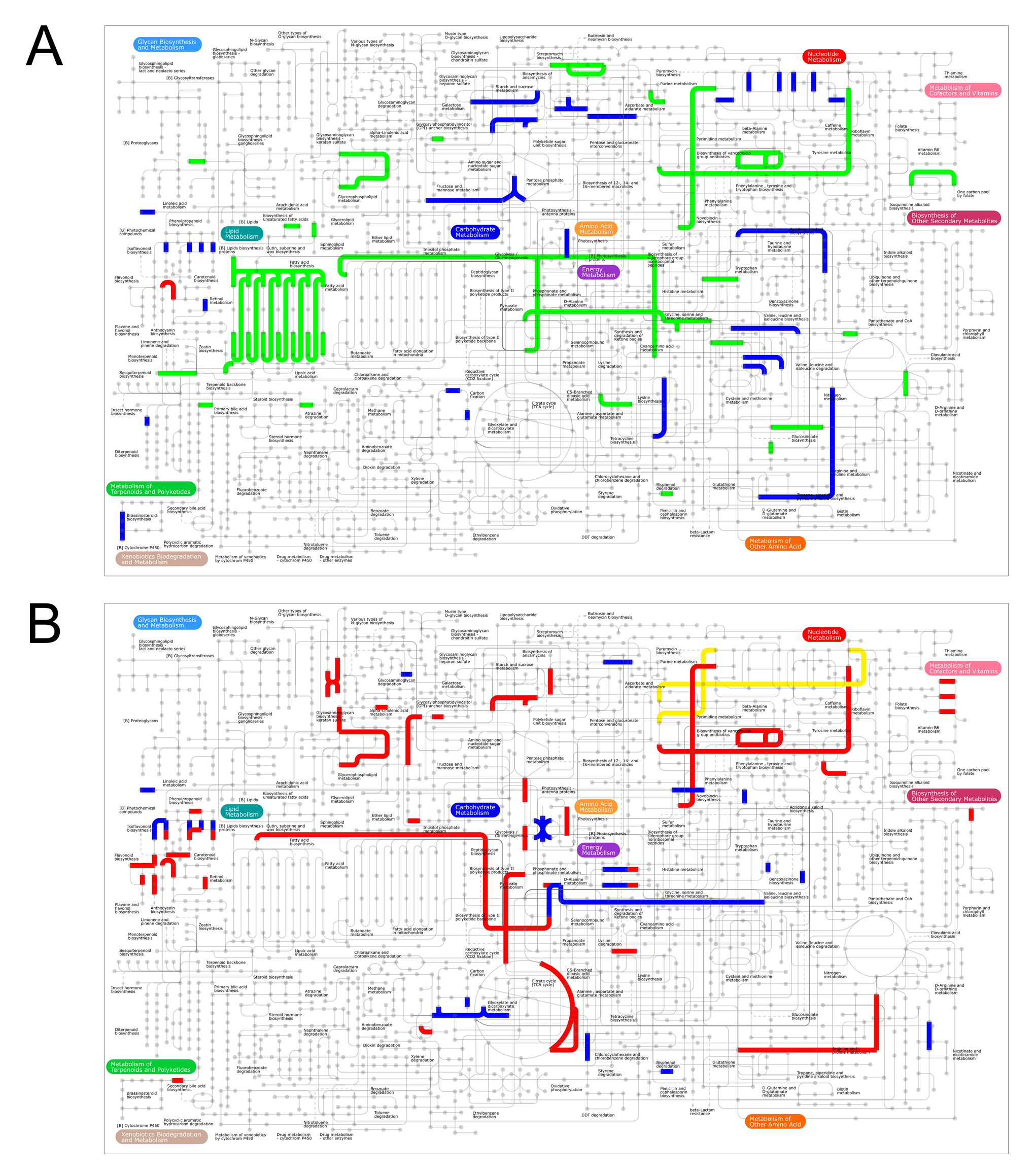

Supplement: Additional file 14: Figure S9. — Interactive pathway analysis of anthocyanin biosynthesis during capitulum development. A, Up-regulated genes after shading. B, Down-regulated genes after shading. [file 12864_2015_1428_MOESM14_ESM.tiff]
